# Tyrosine 23 Phosphorylation-Dependent Cell-Surface Localization of Annexin A2 Is Required for Invasion and Metastases of Pancreatic Cancer

**DOI:** 10.1371/journal.pone.0019390

**Published:** 2011-04-29

**Authors:** Lei Zheng, Kelly Foley, Lanqing Huang, Ashley Leubner, Guanglan Mo, Kelly Olino, Barish H. Edil, Masamichi Mizuma, Rajni Sharma, Dung T. Le, Robert A. Anders, Peter B. Illei, Jennifer E. Van Eyk, Anirban Maitra, Daniel Laheru, Elizabeth M. Jaffee

**Affiliations:** 1 The Sidney Kimmel Comprehensive Cancer Center, Johns Hopkins University School of Medicine, Baltimore, Maryland, United States of America; 2 Department of Oncology, Johns Hopkins University School of Medicine, Baltimore, Maryland, United States of America; 3 The Skip Viragh Center for Pancreatic Cancer, Johns Hopkins University School of Medicine, Baltimore, Maryland, United States of America; 4 Department of Pathology, Johns Hopkins University School of Medicine, Baltimore, Maryland, United States of America; 5 The Sol Goldman Pancreatic Cancer Center, Johns Hopkins University School of Medicine, Baltimore, Maryland, United States of America; 6 Department of Surgery, Johns Hopkins University School of Medicine, Baltimore, Maryland, United States of America; 7 Graduate Program in Cellular and Molecular Medicine, Johns Hopkins University School of Medicine, Baltimore, Maryland, United States of America; 8 Department of Medicine, Johns Hopkins University School of Medicine, Baltimore, Maryland, United States of America; 9 The Johns Hopkins Bayview Proteomics Center, Johns Hopkins University School of Medicine, Baltimore, Maryland, United States of America; University of Pennsylvania, United States of America

## Abstract

The aggressiveness of pancreatic ductal adenocarcinoma (PDA) is characterized by its high metastatic potential and lack of effective therapies, which is the result of a lack of understanding of the mechanisms involved in promoting PDA metastases. We identified Annexin A2 (ANXA2), a member of the Annexin family of calcium-dependent phospholipid binding proteins, as a new molecule that promotes PDA invasion and metastases. We found ANXA2 to be a PDA-associated antigen recognized by post-treatment sera of patients who demonstrated prolonged survival following treatment with a PDA-specific vaccine. Cell surface ANXA2 increases with PDA development and progression. Knockdown of ANXA2 expression by RNA interference or blocking with anti-ANXA2 antibodies inhibits *in vitro* invasion of PDA cells. In addition, post-vaccination patient sera inhibits *in vitro* invasion of PDA cells, suggesting that therapeutic anti-ANXA2 antibodies are induced by the vaccine. Furthermore, cell-surface localization of ANXA2 is tyrosine 23 phosphorylation-dependent; and tyrosine 23 phosphorylation is required for PDA invasion. We demonstrated that tyrosine 23 phosphorylation resulting in surface expression of ANXA2 is required for TGFβ-induced, Rho-mediated epithelial-mesenchymal transition (EMT), linking the cellular function of ANXA2 which was previously shown to be associated with small GTPase-regulated cytoskeletal rearrangements, to the EMT process in PDA. Finally, using mouse PDA models, we showed that shRNA knock-down of *ANXA2*, a mutation at tyrosine 23, or anti-ANXA2 antibodies, inhibit PDA metastases and prolong mouse survival. Thus, ANXA2 is part of a novel molecular pathway underlying PDA metastases and a new target for development of PDA therapeutics.

## Introduction

Pancreatic ductal adenocarcinoma (PDA) remains a lethal cancer with an overall 5-year survival rate of <5% [1]. Inability to diagnose early, high metastatic potential, and drug resistance account for its low survival rate. Although it is well-established that the pathogenesis of PDAs follows stepwise stages that display increasing cellular atypia and accumulate clonal mutations or aberrant expression of oncogenes or tumor suppressor genes such as *K-Ras*, *p16*, *p53*, *and DPC4/SMAD4* [2], drugs that target these molecular abnormalities have not yet translated into improved clinical responses [3]. The aggressive nature of PDA is featured by its high incidence of metastases at the time of initial diagnosis and high incidence of early metastases following surgical resection. However, little is known about the molecular mechanisms underlying its invasion and metastatic processes. A better understanding of these mechanisms is essential for the development of innovative and improved treatments for PDA.

Cancer immunotherapy treatment approaches are under development for PDA. We developed an allogeneic, granulocyte-macrophage colony-stimulating factor (GM-CSF) secreting PDA vaccine for patients with PDA[4], [5], [6]. Phase I and II trials evaluating this vaccine in patients with resected PDA demonstrated both clinical and immunologic responses[4], [5]. This immunotherapy approach provided immunized lymphocyte reagents for identifying novel PDA antigens that are currently being tested as targets for PDA therapy[7], [8]. The potential therapeutic targets identified so far have also provided important clues for the study of molecular mechanisms underlying PDA development and metastases. Here we report the utilization of a functional proteomic approach that identified Annexin A2 (ANXA2) as a new candidate PDA target of the immune response. In addition, we show that tyrosine 23 phosphorylation-dependent cell-surface localization of Annexin A2 is required for epithelial to mesenchymal transition, invasion, and metastases formation of PDAs.

## Results

### Identification of ANXA2 as a new candidate PDA tumor-associated antigen and biomarker

We used immunized sera from two subjects who demonstrated both evidence of post-vaccination cellular immune responses and prolonged disease-free survival (DFS) and overall survival (OS) in a phase II study of a GM-CSF secreting whole cell PDA vaccine [4] to screen whole cell extracts from the PDA vaccine cell lines which served as the proteome. Protein extracts were separated by a two-dimensional electrophoresis (2-DE); and immunoblot analysis was performed to compare antigen recognition by pre- and post-vaccination sera. Proteins recognized by post-vaccination sera relative to pre-vaccination sera were identified by mass spectrometry. ANXA2 was a protein identified on the post-vaccination sera immunoblots of both patients evaluated. To further evaluate the prevalence of post vaccination humoral responses to ANXA2, purified recombinant ANXA2 was used to screen pre-vaccination and post-vaccination sera from 16 additional patients treated in this phase II study by both ELISA and western blot (**Figure S1**). Vaccine induced anti-ANXA2 antibodies, measured by a sandwich ELISA, were detected in 6 of 7 patients who demonstrated a DFS greater than 36 months [4], and only in 1 of the other 9 patients who did not demonstrate long-term DFS (**Table 1**). These data provide the first evidence that ANXA2 is an antibody target of immune responses against PDA.

**Table 1:** Correlation between vaccine-induced anti-ANXA2 antibody responses and patients' disease-free survival.

Patient ID*	Disease Status	Disease-Free SurvivalTime (Mo)**	Increased Anti-ANXA2 antibody response(ELISA)
**3.009**	**Disease free**	**>36**	**+**
**3.010**	**Disease free**	**>36**	**-**
**3.012**	**Disease free**	**>36**	**+**
**3.016**	**Disease free**	**>36**	**+**
**3.027**	**Disease free**	**>36**	**+**
**3.028**	**Disease free**	**>36**	**+**
**3.031**	**Disease free**	**>36**	**+**
**3.041**	**Recurrent**	**<36**	**-**
**3.023**	**Recurrent**	**<36**	**-**
**3.032**	**Recurrent**	**<36**	**-**
**3.025**	**Recurrent**	**<36**	**+**
**3.033**	**Recurrent**	**<36**	**-**
**3.039**	**Recurrent**	**<36**	**-**
**3.001**	**Recurrent**	**<36**	**-**
**3.004**	**Recurrent**	**<36**	**-**
**3.037**	**Recurrent**	**<36**	**-**

*Patients underwent surgical resection of PDA and were treated with a GM-CSF secreting PDA vaccine in a phase II clinical trial.

**Disease-free survival is defined as the time from surgery until disease recurrence or until the last follow up on April 21, 2008; Mo, month.

ANXA2 is reported to be overexpressed in a variety of cancers including PDA when compared with normal tissues[9]. However, ANXA2 is normally a cytoplasmic and lumenal residing protein in pancreatic tissue, and previous studies[9] have not determined whether cell surface ANXA2 expression is a dominant pattern in PDA tissues. Therefore, we analyzed the location of ANXA2 expression by immunohistochemistry (IHC) in the resected tumors from 52 of the 60 patients treated in our Phase II study for whom specimens were available for staining. We found that normal pancreatic ductal epithelial cells show weak cytoplasmic and lumenal staining by IHC, whereas cell-surface localized ANXA2 increases with progression from PanIN lesions to invasive PDA (**Figure S2**). Specifically, 39 (75%) of 52 fresh pancreatic tumor tissue samples tested have increased cell surface expression of ANXA2 (**Figure S2**). These data provide further support that ANXA2 surface expression is associated with PDA development and as such may serve as an immunologic target.

### Inhibition of ANXA2 suppresses the *in vitro* invasion of PDA cells

**Figure 1:**
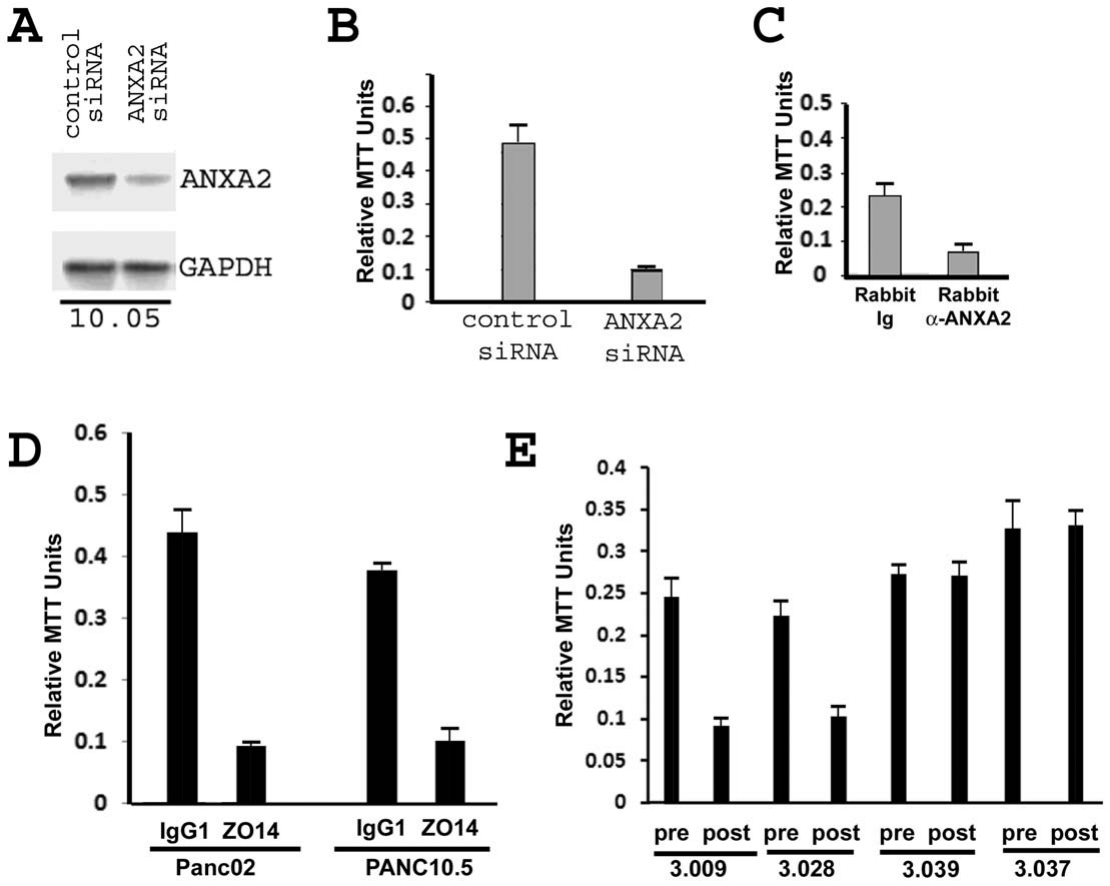
RNA interference, anti-ANXA2 antibodies, and vaccine-induced sera inhibit ANXA2-mediated PDA invasion *in vitro*. **A.** Western blot analysis showing that *ANXA2* siRNA inhibits expression of ANXA2 in a PDA cell line. Whole cell extracts from Panc10.05 treated with control siRNA and *ANXA2* siRNA, respectively, were blotted by rabbit polyclonal anti-ANXA2 antibody (upper panel) and by rabbit polyclonal anti-GAPDH antibody (lower panel). **B.** *In vitro* invasion assay showing that *ANXA2* siRNA inhibits the invasion capacity of the 10.05 PDA cell line. Invaded cells were measured by MTT assays and normalized to total cell numbers. **C.** Polyclonal anti-ANXA2 antibodies inhibit the invasion capacity of Panc10.05 cells. **D.** Mouse anti-ANXA2 monoclonal antibodies (mAb) inhibit the invasion capacity of mouse Panc02 and human Panc10.5 cells. For **C** and **D**, rabbit anti-ANXA2 antibody, rabbit control Ig, mouse anti-ANXA2 mAb (clone: ZO14), or mouse isotype control IgG1 was added into the culture media at a final concentration of 25 µg/ml throughout the *in vitro* invasion assays, respectively. **E.** Only post-vaccination sera from patients (3.009 and 3.028) who demonstrated anti-ANXA2 antibody responses, but not from patients (3.037 and 3.039) who did not demonstrate anti-ANXA2 antibody responses, inhibit invasion of Panc10.05 cells. Pre- and post- vaccination sera were added to the culture media at a ratio of 1∶50. Triplicate experiments were done for **B**–**E**.

We next investigated whether the cell surface localization of ANXA2 plays a biologic role in facilitating PDA invasion. ANXA2 has been reported to bind membrane-associated phospholipids and have diverse cellular functions including plasminogen activation, fibrinolysis, membrane transport, cytoskeleton rearrangement, angiogenesis, cell adhesion and migration. ANXA2 also functions as a high-affinity receptor for multiple extracellular ligands that have been implicated in cancer development, invasion, and metastases [10], [11], [12], [13]. To directly test whether ANXA2 is involved in PDA invasion, ANXA2 expression was knocked down in PDA cells by RNA interference (**Figure 1A**). Knock-down of *ANXA2* suppressed the *in vitro* invasion of PDA cells in a Boyden chamber assay (**Figure 1B** **and Figure S3**). The induction of antibodies against ANXA2 that is observed in vaccinated patients with prolonged DFS (**Table 1**) suggests that anti-ANXA2 antibodies may have a direct anti-tumor effect. We therefore tested both rabbit polyclonal and mouse monoclonal anti-ANXA2 antibodies and found that they can specifically inhibit *in vitro* invasion of PDA cells (**Figure 1C,D**). Moreover, sera from immunized patients who demonstrated a post-vaccination response to ANXA2 similarly inhibited *in vitro* invasion of PDA cells (**Figure 1E**). The data presented so far link increasing cell surface expression of ANXA2 with PDA invasion capability and suggests that vaccine-induced antibody responses may inhibit this aspect of PDA progression. However, the mechanism by which ANXA2 mediated PDA invasion occurs has yet to be explored. Interestingly, the invasive capacity of PDA cells is not correlated with their proliferative rate suggesting an independent mechanism (**Figure S3**). To uncover other regulatory mechanisms that account for the invasion capacity of PDA cells, we further examined the sub-cellular localization of ANXA2 in various PDA cell lines by fluorescent staining with anti-ANXA2 antibodies (**Figure S4**). ANXA2 is predominantly localized to the cell membrane in all 8 PDA cell lines found to have high invasion capacity, whereas ANXA2 is present predominantly in the cytoplasm of cell lines with low invasion capacity (**Figure S4 and** **Table S1**). This data further support a role for ANXA2 translocation from the cytosol to the cell surface/membrane in enhancing PDA cell invasion.

### Phosphorylation of ANXA2 at Tyr23 promotes the cell-surface localization of ANXA2 and the invasion capacity of PDA cells

**Figure 2:**
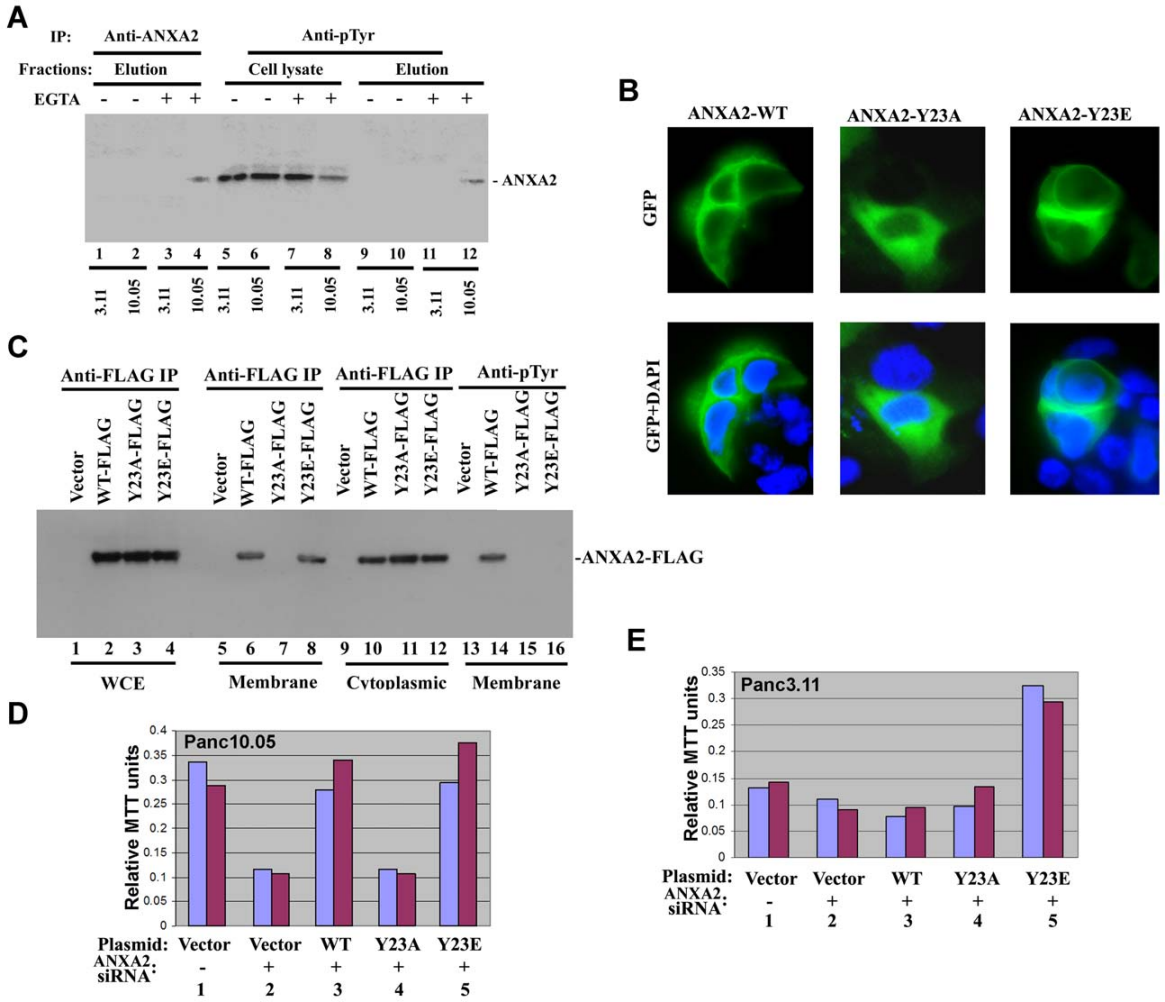
Tyr23 phosphorylation-dependent membrane/cell-surface localization of ANXA2 is required for *in vitro* invasion of PDA cells. **A.** Panc10.05 and Panc3.11 cells were either incubated with EGTA containing buffer or EGTA-free buffer. Two different elutions from two different PDA cell lines, as indicated, were immunoprecipitated by anti-ANXA2 antibodies (lanes 1–4) or anti-phosphotyrosine (anti-pTyr) antibodies (lanes 9–12). After elution, the two PDA cell lines were lysed and the lysates were immunoprecipated by anti-pTyr antibodies (lanes 5–8). **B.** GFP-tagged ANXA2 in Panc10.05 cells. Upper panels: GFP signals; lower panels: overlapped images of GFP signals and DAPI staining of nuclei. **C.** FLAG-tagged ANXA2 expression in Panc10.05 cells transfected with the pcDNA-based plasmid vector alone (lanes 1,5,9,13), the plasmid carrying ANXA2^WT^-FLAG (lanes 2,6,10,14), the plasmid carrying ANXA2^Y23A^-FLAG (lanes 3,7,11,15), or the plasmid carrying ANXA2^Y23E^-FLAG (lanes 4,8,12,16). Whole cell extracts (WCE) (lanes 1–4), cell membrane fractions (lanes 5–8, 13–16), or cytoplasmic fractions (lanes 9–12) were isolated by biochemical fractionation from the Panc10.05 PDA cells, respectively, and immunoprecipitated using either anti-FLAG M2 antibodies (lanes 1–12) or anti-phosphotyrosine antibodies (anti-pTyr) (lanes 13–16). The immunoprecipitates were blotted using anti-FLAG M2 antibodies. **D.** *In vitro* invasion of Panc10.05 cells. **E.** *In vitro* invasion of Panc3.11 cells. For both **D** and **E**, cells were transfected with the empty pcDNA-based plasmid vector (lanes 1,2), the plasmid carrying ANXA2^WT^-FLAG (lane 3), the plasmid carrying ANXA2^Y23A^-FLAG (lane 4), or the plasmid carrying ANXA2^Y23E^-FLAG (lane 5). Lane 1 was also cotransfected with the scramble siRNA control. Lanes 2–5 were also cotransfected with *ANXA2* siRNA duplex. Results of duplicate experiments are shown.

ANXA2 is a substrate for Src kinase, which phosphorylates ANXA2 at Tyr23 both *in vivo* and *in vitro* [10], [11], [12], [13], and Tyr23 phosphorylation has been suggested to be important for normal cell scattering and branching morphogenesis[14], [15]. ANXA2 is also reported to be tyrosine-phosphorylated when it localizes to the cell surface under stress [16]. Since malignant cells often mimic normal cells that have been subjected to a variety of stress stimuli, we postulate that ANXA2 is translocated to the cell surface as a tyrosine-phophorylated protein during tumorigenesis as well. To test this, we eluted the cell surface fraction of ANXA2 from Panc10.05 PDA cells, which have cell surface localization of ANXA2 (**Figure S4 and** **Table S1**), and found the cell surface fraction of the ANXA2 protein is in fact a tyrosine-phosphorylated protein (**Figure 2A**). In contrast, ANXA2 could not be eluted from the cell surface of Panc 3.11 cells, a PDA cell line that demonstrated cytoplasmic localization of ANXA2 (**Figure S4**). To test whether phosphorylation of ANXA2 at Tyr23 is important for its localization to the PDA cell surface, we generated a panel of plasmids expressing either wild-type ANXA2 (ANXA2^WT^), the ANXA2 mutant protein (ANXA2^Y23A^) in which Tyr23 was altered to an alanine residue making a non-phosphorylatable mutant, or the ANXA2 mutant protein (ANXA2^Y23E^) in which Tyr23 was altered to a glutamic acid residue mimicking constitutive phosphorylation. When Panc10.05 cells were transfected with these plasmids expressing ANXA2 tagged by GFP [17], ANXA2^WT^-GFP and ANXA2^Y23E^-GFP localized predominantly to the cell surface of PDA cells. In contrast, ANXA2^Y23A^-GFP localized to the cytoplasm (**Figure 2B**). These results were further confirmed by using a lentivirus to constitutively express ANXA2^WT^, ANXA2^Y23A^, or ANXA2^Y23E^ in the PDA cells (**Figure S4**). Taken together, these data demonstrate that phosphorylation at Tyr23 results in the localization of ANXA2 at the cell surface.

To determine whether the change in ANXA2 localization that occurs as a result of Tyr23 phosphorylation affects the invasion capacity of PDA cells, a set of plasmids that express exogenous FLAG-tagged ANXA2 including ANXA2^WT^-FLAG, ANXA2^Y23A^-FLAG, and ANXA2^Y23E^-FLAG were developed. These vectors are RNA interference resistant because of silent mutations within the siRNA target site. Panc10.05 PDA cells transfected with these plasmids were fractionated into cytoplasmic and cell membrane fractions (**Figure S4**). We first confirmed that only ANXA2 ^WT^-FLAG and ANXA2^Y23E^-FLAG, but not ANXA2^Y23A^-FLAG, localize to the cell membrane fraction (**Figure 2C**). As expected, ANXA2 ^WT^-FLAG protein is tyrosine phosphorylated in the cell membrane fraction. Next, we found that co-transfection of the pcDNA plasmid expressing ANXA2^WT^-FLAG or ANXA2^Y23E^-FLAG, but not ANXA2^Y23A^-FLAG, with the siRNA (to inhibit endogenous ANXA2), reversed siRNA-mediated inhibition of invasion of Panc10.05 cells (**Figure 2D**). However, in cells with low invasion capacity and only cytoplasmic localization of ANXA2, such as Panc3.11 (**Table S1**), co-transfection with ANXA2^Y23E^-FLAG bypasses the phosphorylation regulatory mechanism by mimicking constitutive phosphorylation and promotes the invasion of Panc3.11 cells (**Figure 2E**). These data suggest that Tyr23 phosphorylated ANXA2 confers PDA invasion capacity.

### ANXA2 contributes to the Epithelial-Mesenchymal Transition of PDA cells

**Figure 3:**
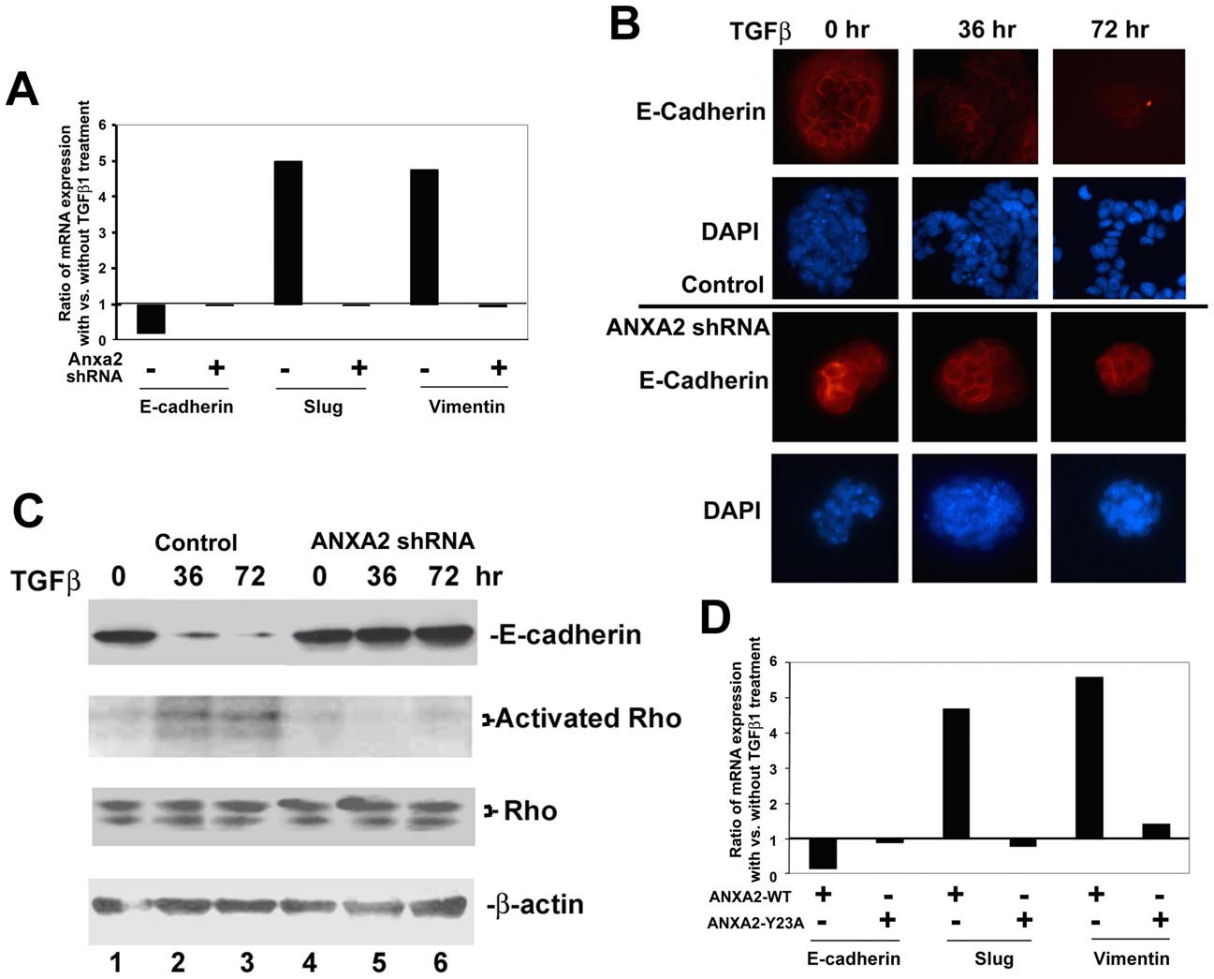
ANXA2 expression and Tyr23 phosphorylation are required for the EMT process in PDA cells. **A.** Quantitative real-time PCR analysis of E-cadherin, slug, and vimentin mRNA expression in Panc10.05 PDA cells with and without knockdown of ANXA2 by shRNA. The relative ratios of mRNA expression with TGFβ1 treatment versus without TGFβ1 treatment are shown. The data were normalized with β-actin expression. **B.** The same pair of PDA cells employed in panel **a** were treated with TGFβ1 for 0, 36, or 72 hours, respectively, and then harvested for immunostaining with anti-E-cadherin antibodies and PE-conjugated secondary antibodies. DAPI was used to stain the nuclei. **C**. The same pair of PDA cells employed in panel **A** were treated with TGFβ1 for 0, 36, or 72 hours, respectively, and then harvested. A fraction of cell extract was used for western blot analysis and was stained with anti-E-caherin, anti-RhoA,B,C, or anti-β-actin antibodies as the internal control, respectively. The remaining cell extract underwent a pull down assay through an affinity column that specifically binds activated, GTP-bound forms of Rho. This was followed by western blot analysis with anti-Rho antibodies. Note that anti-Rho antibodies recognize Rho A, B and C, whose molecular weights are slightly different, resulting in two bands on the gel. Control designates the cells with control shRNA; *ANXA2* shRNA designates the cells with *ANXA2* shRNA. **D.** Quantitative real-time PCR analysis of E-cadherin, slug, and vimentin mRNA expression in a pair of Panc10.05 PDA cell lines infected with lentivirus expressing wild-type ANXA2 (ANXA2-WT) or Y23A mutated ANXA2 (ANXA2-Y23A), respectively. The relative ratios of mRNA expression with TGFβ1 treatment (indicated with +) versus without TGFβ1 treatment (indicated with -) are shown. The data were normalized with β-actin expression.

Phosphorylated ANXA2 plays a role in cell scattering in normal morphogenesis processes[14], [15]. Our data so far support a role for phosphorylated ANXA2 in PDA invasion. The epithelial to mesenchymal transition (EMT) regulates the normal morphogenic process during embryonic development and tissue restructuring, and the initial steps of invasion and metastases are suggested to mimic EMT[18]. Therefore, we sought to determine whether ANXA2 is required for the EMT in PDA cells. EMT is characterized by the suppression of the transcription of epithelial markers such as E-cadherin and induction of mesenchymal markers such as slug and vimentin. ANXA2 has been reported to mediate TGFβ-activated EMT during the process of cardiac valve development [19]. In addition, TGFβ is reported to induce EMT in cultured PDA cells [20], [21]. To examine whether ANXA2 has a direct role in the EMT process of invading PDA cells, a lentiviral vector containing *ANXA2* shRNA was used to achieve long-term suppression of ANXA2 in PDA cells (**Figure S3**). Real-time PCR analysis showed that E-cadherin was suppressed, whereas slug and vimentin were induced during TGFβ-induced EMT in Panc10.05 cells with control shRNA, but not in those infected with *ANXA2* shRNA (**Figure 3A**). In addition, E-cadherin protein expression was suppressed in TGFβ-treated cells with control shRNA, but remained unchanged in TGFβ-treated cells that also expressed *ANXA2* shRNA (**Figure 3B,C**). As predicted, PDA cells without *ANXA2* shRNA lose their cell-cell adhesion phenotype and scatter around the culture slip following TGFβ treatment, reminiscent of an EMT pattern (**Figure 3B**). Although ANXA2 has not yet been shown to be involved in SMAD4-mediated EMT, it has been shown to be involved in Rho (small GTPases)-mediated cell detachment, a trait of EMT [15]. Therefore, we also evaluated whether Rho mediates ANXA2-associated EMT in PDA and found that Rho activation is not detected in PDA cells with shRNA inhibition of ANXA2 following TGFβ treatment (**Figure 3C**). These results demonstrate that loss of ANXA2 expression leads to loss of TGFβ-Rho-mediated EMT in PDA cells.

We next examined whether Tyr23 phosphorylation is important for ANXA2-mediated EMT in PDA cells. We observed that the endogenous ANXA2 no longer localizes to the cell surface in the cells expressing the tyrosine site loss variant ANXA2^Y23A^ (**Figure S4**). In addition, transfection with ANXA2^Y23A^-FLAG inhibits the invasion of Panc10.05 cells (**Figure S4**), suggesting that ANXA2^Y23A^ has a dominant negative effect. Consistent with published data [22], we found that ANXA2^Y23A^ can still bind to its partner S100A10/p11 [23] in the cytosol, but not in the cell membrane (**Figure S5**). Therefore, overexpressed, cytoplasmic-localized ANXA2^Y23A^ may sequester all of the S100A10/p11 in the cytosol, thereby conferring a dominant negative effect. Therefore, taking advantage of the dominant negative effect of ANXA2^Y23A^, and employing this ANXA2^Y23A^ mutant, we further demonstrated that EMT is induced by TGFβ in cells expressing ANXA2^WT^, but not in cells expressing ANXA2^Y23A^ (**Figure 3D**). Thus, these data further demonstrate that the Tyr23 phosphorylation of ANXA2 promotes the EMT of PDA cells and is one conceivable mechanism by which ANXA2 localizes to the PDA cell membrane and confers the potential for PDA cells to invade.

### Expression and tyrosine phosphorylation of ANXA2 are required for PDA metastases formation *in vivo*

**Figure 4:**
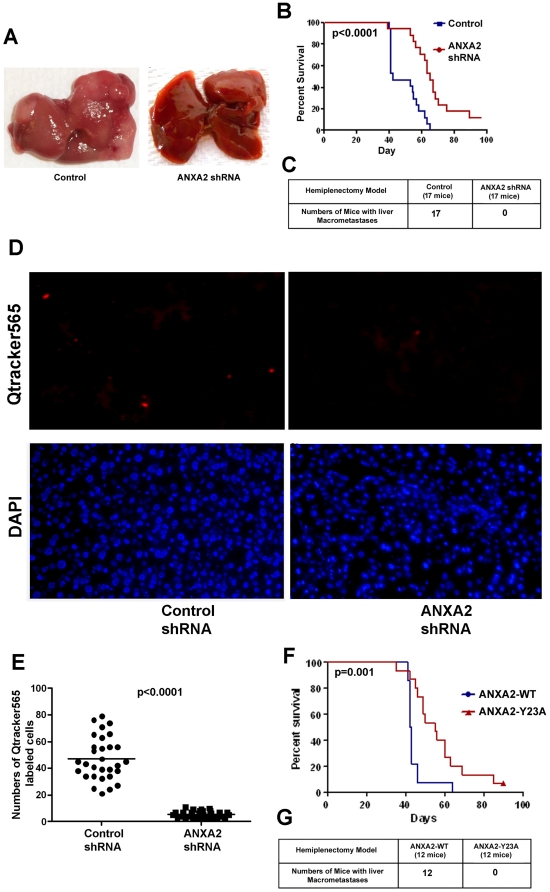
shRNA-knockdown of ANXA2 or a mutation at Tyr23 inhibits PDA metastases and prolongs mouse survival. **A.** Livers from representative mice receiving intrasplenic injections of mouse PDA cells infected with a lentivirus carrying either control shRNA or *ANXA2* shRNA. **B.** Kaplan-Meier curves comparing the survival of mice receiving Panc02 cells carrying either control shRNA or *ANXA2* shRNA. A log-rank analysis on Day 90 showed that inhibition of ANXA2 expression with shRNA significantly prolonged survival (p<0.0001). **C.** Summary of the number of mice developing metastases in both control and *ANXA2* shRNA groups under macroscopic inspection. None of the mice in the *ANXA2* shRNA group formed liver macro-metastases. **D.** Shown are representative frozen sections of mouse liver imaged under fluorescent microscope assessing Qtracker565-positive cells and DAPI-stained nuclei. Panc02 cells labeled with Qtracker565 (Invitrogen) were injected into the mouse splenic bed followed by hemisplenectomy. Three mice in each group were injected with either Panc02 carrying the control shRNA or Panc02 carrying the *ANXA2* shRNA. Five days later, mice were euthanized and 10 frozen sections that cross each liver were stained with DAPI for nuclei and then examined under fluorescent microscope. In each frozen section, five 20x fields were randomly chosen to score the numbers of Qtracker565-positive cells. **E.** The sum of five fields of each section was considered to be one individual data set and a comparison was made between the control shRNA and the *ANXA2* shRNA expressing PDA tumor cells. The result shows that significantly fewer Qtracker565–positive cells were found in the livers from the *ANXA2* shRNA group as compared with the control shRNA group (p<0.0001). **F.** Kaplan-Meier curves comparing the survival of mice receiving Panc02 cells expressing either ANXA2-WT or ANXA2-Y23A. A log-rank analysis showed that expression of the Tyr23 phosphorylation-deficient mutant of ANXA2 significantly prolonged survival (p = 0.001). **G.** Summary of the number of mice developing metastases in both ANXA2-WT and ANXA2-Y23A groups. None of the mice in the ANXA2-Y23A group formed liver macro-metastases.

Local invasion by tumor cells is a known step in the process of metastasis. Our data demonstrate that ANXA2 facilitates invasion of PDA cells *in vitro*. We therefore employed a transplantable murine pancreatic cancer model of metastases (**Figure S6**) to evaluate the role of ANXA2 expression, phosphorylation, and cell surface localization in the PDA metastasis process *in vivo*. In this model, 100% of the mice die with liver metastases at approximately 4-6 weeks (**Figure 4A**) after splenic injection of 2×10^6^ Panc02 murine PDA cells. Panc02 cells infected with a GFP expressing lentivirus carrying shRNA specific for *ANXA2* knockdown or control shRNA were sorted for GFP-positive cells by FACS. After confirming the knockdown of ANXA2 expression (**Figure S3**), 2×10^6^ cells were injected into the splenic bed prior to performing a hemisplenectomy, and mice were monitored for survival. Mice injected with Panc02 cells expressing the *ANXA2* shRNA survived significantly longer than mice injected with Panc02 cells expressing control shRNA (p<0.0001) (**Figure 4B**). Necropsy was performed on all mice that died, as well as those that still survived to day 90 following tumor implantation. Macroscopic inspection showed that 17/17 mice in the control group, but no mice (0/17) in the *ANXA2* shRNA group developed liver macro-metastases (**Figure 4C**). Additional pathologic analyses indicated that mice in the *ANXA2* shRNA group died due to tumor formed at the splenic bed (local regional tumor growth) (**Figure S7**). To confirm that PDA cells carrying *ANXA2* shRNA are not able to seed the liver, 1×10^6^ Panc02 cells labeled with Qtracker 565 were injected at the splenic bed. Five days later, frozen sections of liver were examined under fluorescent microscope for Qtracker 565-labeled cells (**Figure 4D**). Significantly fewer Qtracker 565-positive cells are seen in the livers of *ANXA2* shRNA mice when compared with the control shRNA group (p<0.0001) (**Figure 4E**).

The ANXA2 protein sequence including Tyr23 is conserved in mammals and its phosphorylation can be induced in non-human mammalian cells [14], [15], [24]. Thus, the hemisplenectomy model was again employed to examine whether Tyr23 phosphorylation of ANXA2 is required for ANXA2-mediated murine PDA metastases. Mice injected with Panc02 cells stably expressing exogenous ANXA2^Y23A^ survived significantly longer than mice injected with Panc02 cells stably expressing exogenous ANXA2^WT^ (p = 0.001) (**Figure 4F**). All 12 mice in the control group whereas 0/12 mice in the ANXA2^Y23A^ group developed liver macro-metastases (**Figure 4G**).

We also used a previously established orthotopic model in which PDAs metastasize to the peritoneum [25] to confirm that ANXA2 can initiate metastasis from a primary tumor in addition to facilitating the seeding of PDA metastases in the liver. This study confirmed that *ANXA2* knockdown does not affect the growth of primary PDAs significantly (**Figure S8**). All mice in the control group developed large peritoneal macro-metastases. In contrast, most mice in the *ANXA2* shRNA group did not develop macro-metastases. In addition, the mean size of metastatic lesions that formed in *ANXA2* shRNA mice, typically manifesting as micro-metastatic foci, was significantly smaller (P = 0.0012) than the control mice (**Figure S8**). IHC analysis of cell surface ANXA2 demonstrated that these micro-metastatic foci were formed by cells that still expressed ANXA2 due to incomplete knockdown by *ANXA2* shRNA (**Figure S8**). Taken together, our data provide strong evidence that Tyr23 phophorylated and cell surface expressed ANXA2 facilitates the metastatic process, and supports ANXA2 as a novel target for PDA therapy development.

### Anti-ANXA2 antibodies suppress PDA metastases *in vivo* and prolong survival in a mouse model of PDA

**Figure 5:**
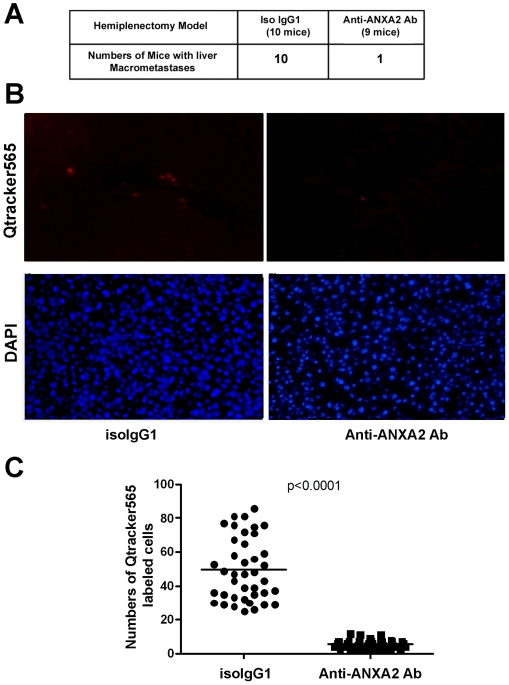
Anti-ANXA2 antibodies inhibit PDA metastases and prolong mouse survival. **A.** Summary of the number of mice developing metastases in both isotype control IgG1 and anti-ANXA2 Ab groups. Only one of the mice in the anti-ANXA2 Ab group formed liver macro-metastases. Mice in the anti-ANXA2 Ab group survived significantly longer than mice in the control IgG group (p = 0.02). **B.** Shown are representative frozen sections of mouse liver (4 mice/group studied) imaged under a fluorescent microscope for Qtracker565-positive cells and for DAPI-stained nuclei as described in **Figure 4D–E**. **C.** The sum of five 20X fields of each section was considered as one individual data set and was analyzed in the statistical comparison between the isotype control IgG1 and the anti-ANXA2 antibody group. The result shows that significantly fewer Qtracker565–positive cells were found in the livers of mice treated with the anti-ANXA2 antibody as compared with the control IgG1 antibody (p<0.0001).

Next, we evaluated whether an inhibitory ANXA2-targeted monoclonal antibody can suppress Panc02 hepatic metastases using the above described liver metastases forming mouse model. Specifically, Panc02 injected mice were treated twice weekly with a mouse monoclonal anti-ANXA2 antibody (shown to inhibit Panc02 invasion *in vitro* in **Figure 1**) until death. All mice (10/10) in the control IgG group whereas only 1/9 mice in the ANXA2 antibody group developed liver macro-metastases (**Figure 5A**). Furthermore, mice treated with the anti-ANXA2 antibody survived significantly longer than mice treated with isotype control IgG (p = 0.02). Similar to the mice receiving *ANXA2* shRNA, mice in the ANXA2 antibody group died due to tumor progression at the splenic bed, suggesting this anti-ANXA2 antibody therapy does not inhibit the growth of primary PDAs. Predictably, mice implanted with Qtracker 565-labeled Panc02 cells and treated with a single intraperitoneal injection of anti-ANXA2 antibody were found to have significantly (p<0.0001) fewer Qtracker 565-positive cells in their livers as compared with mice given the control mAb (**Figure 5B,C**). These data further support ANXA2 as a target for therapeutic intervention of metastases.

## Discussion

ANXA2 was brought to our attention as a target of vaccine induced immune responses identified on a proteomic screen of PDA proteins using immunized sera from patients who demonstrated prolonged DFS. A previously published study links ANXA2 overexpression in pancreatic cancer tissue with rapid recurrence after gemcitabine adjuvant chemotherapy in postoperative patients [26]. In this study, we demonstrate three new findings that elucidate the role of ANXA2 in PDA invasion and metastases. First, localization of ANXA2 expression on the cell surface is required for PDA invasion *in vitro* and metastases formation *in vivo*. Second, Tyr23-phosphorylation is required for localization of ANXA2 on the PDA cell surface and subsequent PDA invasion and metastases formation. Importantly, knockdown of ANXA2 or inhibition with ANXA2 antibody therapy inhibits the metastatic process. Third, loss of ANXA2 expression or phosphorylation at Tyr23 leads to loss of TGFβ-Rho-mediated EMT in PDA cells. Taken together, these findings identify a new role for phosphorylated ANXA2 in mediating PDA cell invasion via Rho-regulated EMT and facilitating PDA metastases.

Our study shows for the first time that cell surface localization of ANXA2, mediated by Tyr23 phosphorylation, enhances PDA cell invasion *in vitro* and PDA metastases to liver *in vivo*. Relevant to our findings, a recent report showed that knockdown of ANXA2 in a PDA cell line inhibits cell migration *in vitro* [27]. We also evaluated a panel of PDA tumor cell lines and 52 fresh tissue specimens and showed the frequent expression of cell surface ANXA2 in primary PDAs, which further supports a role for ANXA2 in PDA progression. Another report demonstrated that the reduction in cell surface ANXA2 expression in response to changes in interferon gamma levels leads to reduced prostate cancer cell invasion[28], suggesting that the cell surface translocation of ANXA2 may be an important cellular process for multiple types of cancers[10].

We also show that ANXA2 phosphorylation is required for TGFβ-induced and Rho-mediated EMT phenotypes of PDA cells. A previous study showed that loss of Smad4 expression in PDA can lead to the aberrant activation of STAT3, which may contribute to the switch in function of TGFβ from a tumor-suppressive to a tumor-promoting EMT pathway in PDA [20]. Currently, knowledge of the EMT process in cancer development is largely limited to its characteristic transcription circuit. It is still unknown how cancer cells functionally undergo cytoskeletal rearrangement to exhibit mesenchymal features, interact with extracellular matrix, and migrate to distant locations when the EMT process is initiated. One of the cellular functions of ANXA2 is its involvement in cytoskeletal rearrangement, which is thought to be mediated by its tyrosine phosphorylation and its interaction with small GTPases [14], [15]. Thus, our data, linking ANXA2 and EMT in PDA, provide a new pathway that may regulate the EMT process that occurs in cancer invasion and metastases.

However, it is still not clear which tyrosine kinases phosphorylate ANXA2 in PDA. A number of tyrosine kinases including Src, insulin receptor, and insulin-like growth factor (IGF)-1 receptor have previously been implicated in regulating the cellular function and cell surface translocation of ANXA2 in normal morphogenesis processes[14], [15], [24]. Further investigation of these tyrosine kinases as mediators of ANXA2 phosphorylation in PDA is therefore warranted to further delineate the mechanism by which ANXA2 becomes phosphorylated in PDA. In addition, inhibitors of Src and IGF-1 receptor are under clinical development for the treatment of various cancers including PDA [29]. Therefore, ANXA2 phosphorylation or its cell surface translocation may also serve as a biomarker for assessing treatment response to these agents. Importantly, blocking ANXA2 by anti-ANXA2 antibodies can inhibit PDA invasion and metastases, suggesting that ANXA2 and its associated pathways are viable therapeutic targets.

We have used two mouse models of PDA that have provided complementary data supporting a role for ANXA2 in PDA progression and metastases formation *in vivo*. Data employing the PDA orthotopic model suggests that ANXA2 confers PDA cells with the invasion and metastasis potential when the PDA cells originate from the primary tumor. Data from the liver metastasis model suggests that ANXA2 also facilitates PDA cell metastasis formation once the PDA cells gain access to the blood supply supporting a metastastic site. Thus, ANXA2 appears to be important for the initial invasion process and for the survival of PDA cells at their metastatic destination. Accumulated evidence has supported the “seed-and-soil” hypothesis that the metastatic “seeds” of a given cancer only take root and grow in certain organs with a welcoming microenvironment or “soil” [30]. Our data support this hypothesis and suggest that ANXA2 facilitates PDA cell invasion and motility locally, but also provides an important signaling pathway linking the PDA cell to the growth signals in the new metastatic environment (the “soil”).

In summary, this study provides *in vivo* evidence supporting ANXA2 as a mediator of PDA invasion and metastases. Additional studies are needed to further elucidate the mechanisms by which ANXA2 interacts with PDA stroma at both the primary and metastatic tumor sites to accomplish these processes. Importantly, this study demonstrated that blocking ANXA2 by anti-ANXA2 antibodies can inhibit PDA metastases, providing the rationale for developing therapeutic agents that target ANXA2.

## Materials and Methods

### Cell lines and tissue culture

The human pancreatic ductal adenocarcinoma (PDA) cell lines were previously described [31], [32]. The human fibroblast cell line was established from human PDA paracancerous tissues. All human PDA cell lines were maintained in RPMI1640 media supplemented with 10% fetal bovine serum (FBS) in a humidified incubator at 37°C and 5% CO_2_. The mouse Panc02 cells were maintained in DMEM media supplemented with 10% FBS in a humidified incubator at 37°C and 10% CO_2_. When indicated, TGFβ1 (R&D Systems) was added in the culture medium at a final concentration of 400 pM. Cells were treated with TGFβ1 for 48 hours before harvest unless otherwise noted. The Panc02 cell line is an established chemical carcinogen induced mouse PDA cell line that originated from the C57Bl/6 mice [33].

### Antibodies

The rabbit polyclonal anti-ANXA2 antibody (H50), mouse monoclonal anti-ANXA2 antibody (clone: ZO14), mouse monoclonal anti-p11 antibody, and the mouse monoclonal anti-E-cadherin antibody were obtained from Santa Cruz Biotechnology, Invitrogen, BD Transduction, and Zymed Laboratories, respectively.

### Human serum

Human sera and pancreatic tumor tissue samples were obtained from patients enrolled in a phase II study of an allogeneic GM-CSF secreting whole cell PDA vaccine. Written informed consent was obtained in compliance with the Johns Hopkins Medical Institution Institutional Review Board (IRB)-approved J9988 protocol with approval number #00-01-13-02 [4]. Serum was collected and stored according to standard procedures.

### Enzyme-Linked Immunosorbent Assay (ELISA)

A previously published ELISA assay was used to detect anti-ANXA2 antibody responses in patients [34]. The calibration curve was generated with the post-vaccination sera from patient 3.009.

### DNA cloning and plasmid constructions

The full-length human *ANXA2* cDNA was obtained by reverse transcription of total RNA purified from Panc10.05 cells, followed by high-fidelity PCR amplification with *ANXA2* primers. The non-complementary region of the reverse primer also contained the FLAG tag sequence. The resultant PCR product of *ANXA2* cDNA was then cloned into the pCR vector (Invitrogen) and was sequenced to confirm no introduction of missense or nonsense mutations. The *ANXA2* cDNA fragment with a C-terminal FLAG tag was further subcloned into the lentiviral vector (LV). In this lentivirus, *ANXA2* is expressed under the control of the EF-1α promoter. For the co-transfection of both plasmids and siRNA, the resultant PCR product of *ANXA2* cDNA with a C-terminal FLAG tag was cloned into the pcDNA3.3 vector (Invitrogen) directly. Y23A and Y23E mutations were created by site-directed mutagenesis according to the manufacturer's manual (Stratagene).

### Plasmid transfection, lentiviral infection, and RNA interference

For plasmid transfection and RNA interference, cells were seeded in multiple 6-well plates to 80% confluence. For each well, 2 µg of pcDNA-based plasmid and/or 40 pmol siRNA duplex, were transfected with lipofectamine 2000 in serum-containing medium according to the manufacturer's manual (Invitrogen). For protein expression analysis, cells were harvested at 48 hours. The *ANXA2* siRNA was synthesized by Ambion, Inc.; the scramble siRNA was purchased from Ambion.

To produce lentivirus expressing *ANXA2*, the plasmid with lentiviral constructs was co-transfected with packaging plasmids into 293T cells as previously described [35]. Lentivirus supernatant was collected at 48 hours. For infection, cells were seeded in multiple 6–well plates to 80% confluence. For each well, 2 ml lentivirus supernatant was added and incubated for 48 hours before cells were harvested.

The lentivirus expressing human *ANXA2* hairpin shRNA was obtained from Open Biosystems. Lentivirus was produced according to the manufacturer's manual. For infection of Panc10.05 cells, 6 ml of viral supernatant was added to adherent cells plated in each 75 cm flask and incubated for 48 hours. Cells from two flasks were sorted by GFP in a FACS cell sorter at 72–96 hours after infection. The cells infected with lentivirus expressing GFP alone were sorted. Total RNA was immediately extracted after cell sorting. The lentivirus expressing mouse *ANXA2* hairpin shRNA was obtained from GeneCopoeia.

### Cell invasion assays

Cell invasion assays were carried out using 96-well Transwell plates with 8-µm pores and reagents in the Cultrex BME Cell Invasion Assay system according to the manufacturer's manual with modification (R&D Systems). For all invasion experiments, 0.5xBME (Basement Membrane Extract) was used to coat the top well and 10% serum containing media was added to the bottom well. To score the cells across the transwell, MTT (3-(4,5-Dimethylthiazol-2-yl)-2,5-diphenyltetrazolium bromide) assays were used as previously described [36]. Relative MTT units representing invasion capacity were measured and normalized by total cell numbers. To assess the spontaneous leakage of cells through the BME-coated transwell, invasion specific controls were also performed by adding serum-free media in the bottom wells. To exclude the effect of such leakage, relative MTT units in the invasion experiments were adjusted by subtracting the MTT values of leaked cells in matched invasion specific controls.

PDA cells were plated at 1×10^4^ cells per well in triplicate. PDA cells were transfected with either *ANXA2*-targeted siRNA or control siRNA prior to plating. Invasion was measured at 48 hours following plating using an MTT readout system.

### Fluorescent immunostaining

Panc10.05 cells were grown on cover slips to 90% confluence and were fixed in 4% paraformaldehyde for 15 min. Cover slips were then treated with PBS containing 0.1% Triton X-100 for 5 minutes followed by washing with PBS. After, cover slips were blocked with 10% normal goat sera in PBS for 1 hour and were then incubated with rabbit anti-ANXA2 antibodies or mouse anti-E-cadherin mAb at a 1∶100 dilution in 10% normal goat sera overnight at 4°C. Following a PBS wash, they were further incubated with FITC-conjugated goat anti-rabbit IgG or PE-conjugated goat anti-mouse IgG (Santa Cruz Biotechnology) at a 1∶200 dilution in 10% normal goat sera at room temperature for 1 hour. They were subsequently washed with PBS containing 0.5% NP-40. Cover slips were mounted in a medium containing DAPI (4′,6-diamidino-2-phenylindole) (Vector Labs) and examined by a fluorescent microscope.

### Immunohistochemistry (IHC)

IHC staining for both human and mouse ANXA2 was performed using a standard protocol on an automated stainer from Leica Microsystems. After deparaffinization and hydration of tissue, heat induced antigen retrieval was performed with EDTA buffer (pH 9.0) for 20 minutes. Incubation with the H50 rabbit anti-AnxA2 antibody (Santa Cruz Biotechnology) at a 1∶100 dilution was followed by secondary antibody incubation from the bond polymer refine detection kit (Leica Microsystems). The reaction was developed using substrate 3,3′-Diamino-benzidine hydrochloride (DAB). All slides were counterstained with hematoxylin. Each area of PDA cells on the entire slide was scored from 0 to 3+ by two clinical pathologists (P.I. and R.A.) independently. Scores of 0 to 3+ measure the different intensities of cell-surface staining of AnxA2, with a score of 0 representing no staining and a score of 3+ representing the strongest staining. The percentage of PDA cells at each score level was estimated. The average score of cell-surface AnxA2 expression was calculated as follows:
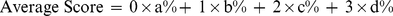
(a%, b%, c%, and d% are the percentages of PDA cells with score 0 to 3, respectively.)

### Whole cell extract and cell fractionation

PDA whole cell extracts were obtained as previously described [37]. In brief, cell pellets were resuspended in Lysis 250 buffer followed by a freeze and thaw process that was performed 3 times. The cell lysate was spun at 15,000 rpm for 10 minutes and supernatant was removed. The protocols to separate membrane and cytoplasmic fractions were adapted from those previously published [38]. EGTA (Ethylene glycol-bis(2-aminoethylether)-*N,N,N',N'*-tetraacetic acid) elution of cell surface AnxA2 followed a previously established procedure [16].

### Immunoprecipitation and immunoblot analysis

Anti-phosphotyrosine antibody conjugated sepharose (P-Tyr-100, Cell Signaling Technology) was used to immunoprecipate tyrosine-phosphorylated proteins. Anti-AnxA2 antibodies were first conjugated to sepharose beads according to the manufacturer's manual (Pierce) prior to being used for immunoprecipitation. Anti-FLAG M2 antibodies-conjugated beads were obtained from Sigma. For anti-p11 immunoprecipitation, cell fractionations were first incubated with anti-p11 antibodies and then with protein A sepharose beads. All immunoprecipitations were done at 4°C overnight, followed by washing with Lysis 250 buffer [37].

After whole cell extracts, cell fractions, or immunoprecipitants were boiled in SDS-sampling buffer, they were loaded on 10% gradient SDS-PAGE (BioRad). The gel was then transferred to a nitrocellulose membrane and blotted with the rabbit anti-AnxA2 polyclonal antibody at a 1∶1000 dilution followed by HRP-conjugated goat anti-rabbit IgG (Amershan Pharmacia) at a 1∶3000 dilution.

Recombinant His6-tagged ANXA2 was expressed in TOP10 *E. coli* and purified on a High-Trap Ni column according to the manufacturer's manual (Amershan Pharmacia). One microgram of purified His6-tagged ANXA2 was loaded on each well of a 10% gradient SDS-PAGE. After transferring to a nitrocellulose membrane, each individual lane was blotted with either pre-vaccination serum or post-vaccination serum at a 1∶1000 dilution. Mouse anti-human IgG antibody (Sigma) was used at a 1∶5000 dilution as the secondary antibody.

Total cellular Rho, or activated Rho which was isolated by incubating the cell extract with an activated Rho affinity binding column (GST-tagged Rho-binding domain of Rhotekin), were detected by western blot with mouse anti-Rho A,B,C mAbs provided by the Rho Activation Assay Kit according to the manufacturer's manual (Upstate).

### Reverse transcription and real-time PCR

RNA was isolated from cells using the RNAEasy kit (Qiagen), and reverse transcribed using the first strand cDNA synthesis kit (Invitrogen). Quantitative real-time reverse transcription-PCR (qRT-PCR) was performed with gene-specific fluorescent TaqMan probes (Applied Biosystems) using an ABI PRISM 7500 Sequence Detection System Instrument and the associated software (Applied Biosystems) following the manufacturer's instructions. Each reaction was performed in triplicate at 2 cDNA dilutions. The standard human β-actin gene (*BACT*; Applied Biosystems) was used to normalize variations in the quantities of input cDNA.

GFP-sorted, shRNA-lentiviral infected cells were subjected to real-time RT-PCR analysis to measure the mRNA expression of E-cadherin, slug, and vimentin. As a negative control, the lentivirus expressing only GFP was also used to infect a control population of Panc10.05 cells. These cells were also sorted for GFP-positive cells for this analysis. One pair of Panc10.05 cell lines, with and without *ANXA2* shRNA, were treated with TGFβ1 for 48 hours before they were harvested for mRNA expression analysis.

### Mouse models of PDA

All animal experiments conformed to the guidelines of the Animal Care and Use Committee of the Johns Hopkins University and animals were maintained in accordance to guidelines of the American Association of Laboratory Animal Care (Approval# MO08M142).

The mouse liver metastasis model was established using a previously described hemispleen injection technique [39], [40]. The transplantable tumor model allows for the assessment of *in vivo* tumor growth following shRNA knockdown of targeted PDA expressed proteins. The spleens of anesthetized female C57Bl/6 mice ages 8 to 10 weeks were divided into two halves and the halves were clipped. Mouse PDA cells (2×10^6^) were injected into the splenic bed (splenic artery and veins) through one hemispleen followed by a flush with the HBSS buffer. After 30 seconds, the splenic vessels draining the injected hemispleen was clipped and the hemispleen was removed. The abdominal wall was sutured, and the skin adapted using wound clips. All mice were followed three times a week for survival. At necropsy, mice were examined macroscopically; and multiple sections of livers and splenic bed injection sites were examined microscopically with H&E staining and, as indicated, anti-ANXA2 IHC.

For survival analysis, mice were treated intraperitoneally with 10 µg anti-ANXA2 monoclonal antibody ZO14 antibodies (Invitrogen) or isotype control IgG1 in 100 µl PBS twice a week starting the day following the hemisplenectomy procedure and continued until death. For the analysis of Qtracker-labeled cells, mice in each group were treated intraperitoneally either with 100 µg anti-ANXA2 monoclonal antibody ZO14 or with 100 µg isotype control IgG1 on the day following the hemisplenectomy procedure and sacrificed 5 days later.

### Statistics

Statistical analysis was performed using software GraphPad Prism 5. Two tailed Mann-Whitney test was used to compare differences between treatment groups. Mouse survival was analyzed by the Kaplan-meier curve and the Log-Rank test. Mouse experimental data from independent experiments were shown in a combined analysis because of the consistency of the results.

## Supporting Information

Table S1 **Cell Surface Localization of Annexin A2 in Cells with Different Invasive Capacities.**
(PDF)

Figure S1 **PDA patients demonstrate vaccine induced ANXA2-specific serologic responses.** **A**. Purified recombinant His6-tagged ANXA2 (His6-ANXA2) on a SDS-PAGE gel stained with coommassie blue. **B**. Purified His6 tagged ANXA2 on a SDS-PAGE gel was western-blotted by pre- and post-vaccination sera. Patients marked by * had antibody induction, which was manifested by stronger signals of ANXA2 on western blot with post-vaccination versus pre-vaccination sera. **C**. Pre- and post-vaccination sera from patients were tested for the presence of anti-ANXA2 antibodies by ELISA using purified recombinant ANXA2-coated plates. Positive antibody induction, which was manifested by a more than 2-fold increase in antibody reaction, was marked by *.
(TIF)

Figure S2 **Cell surface expression of ANXA2 is increased in the majority of human PDAs.** The pattern of ANXA2 expression was analyzed by IHC in 52 of 60 resected tumors from patients treated in a Phase II study for whom specimens were available for staining. Representative IHC staining of ANXA2 in these human PDAs is shown (panels **A**, **B**, **C**). Cell-surface expression of ANXA2 was semiquantitated using a score of 0 to 3, with a score of 0 representing no staining and a score of 3 representing the strongest staining. Normal pancreatic duct, PanINs and PDA are indicated. Cytoplasmic and luminal staining was excluded from scoring. Shown is the intensity of ANXA2 expression on the cell surface. PDA cells vary in their ANXA2 expression level within the same tumor tissue (panel **C**). To account for expression variability within each specimen, the percentage of PDA cells at each score level was estimated and the average score of each PDA tissue was calculated by multiplying each score by their percentages (see Supporting Information Materials and Methods). As expected, none of the normal appearing ductal epithelial cells within the resected tumor masses express 3+ ANXA2 and few express 2+ ANXA2 (panel **B**). An average score of 1.5 or above was considered representative of increased cell surface expression of ANXA2 in the tumor tissue.
(TIF)

Figure S3 ***ANXA2*** **shRNA inhibits the invasion capacity of multiple human and mouse PDA cell lines and PDA cell invasion potentials vary among different PDA cell lines and do not correlate with their proliferative rates.** **A.** Human Panc2.03 PDA cells or mouse Panc02 PDA cells were infected with lentivirus carrying the shRNA specific for human or mouse *ANXA2* or lentivirus carrying the control shRNA. Invaded cells were measured by MTT assays and normalized by total cell numbers. Triplicate experiments were done for control shRNA and *ANXA2* shRNA, respectively. **B.** Panc10.05 (lanes 1,3) or Panc02 cells (lanes 2,4) infected with lentivirus either carrying shRNA specific for human or mouse ANXA2 knockdown (lanes 3 and 4, respectively) or carrying control shRNA (lanes 1,2) were sorted for GFP-positive cells by FACS with one aliquot of cells saved for analysis of ANXA2 expression prior to each experiment. A representative western blot using the rabbit anti-ANXA2 antibody and the rabbit anti-GAPDH antibody (control) is shown. **C.** A panel of PDA cell lines derived from primary resected tumors were evaluated in an *in vitro* invasion assay. Of 11 PDA cell lines tested, 8 have higher and 3 have lower invasion capacity (**Table S1**). Shown are average MTT units on three parallel experiments normalized to total cell numbers. **D.** Expression of ANXA2 in each PDA cell line demonstrated by immunoblot analyses with anti-ANXA2 polyclonal antibody. Lanes 1–11 correspond to human PDA cell lines: Panc01.28, Panc10.05, Panc2.8, Panc2.03, Panc4.03, PancTS0129, Panc3.11, Panc2.13, Panc6.03, Panc9.3.96, and Panc2.43, respectively; lane 12, human pancreatic para-cancerous fibroblast cells. Expression of ANXA2 is slightly lower in cells with lower invasion capacity and slightly higher in those with higher invasion capacity, suggesting that over expression of ANXA2 in PDAs may contribute to, but does not entirely explain the range of invasion potential of these PDA cell lines. **E.** Growth curves of selected PDA cell lines measured by MTT assays. Note that Panc3.11 cells with low invasive capacity grow similarly to Panc10.05 or Panc2.8 with high invasive capacity.
(TIF)

Figure S4 **PDA cell invasion potential correlates with ANXA2 surface localization on PDA cells and PDA cells expressing the exogenous ANXA2-Y23A mutant have reduced surface localization and invasion potential** ***in vitro*** **.** **A**. Fluorescent immunostaining shows predominant cell surface localization of ANXA2 in representative cells with higher invasion capacity (Panc2.43, Panc2.03), but not in cells with lower invasion capacity (human fibroblast, Panc3.11, Panc9.3.96). See supplemental Figure 6 for Panc10.05 cells. FITC indicates the images of immunostaining with rabbit anti-ANXA2 polyclonal antibody and FITC-conjugated secondary antibody. FITC+DAPI indicates the overlapped images of FITC staining of ANXA2 and DAPI staining of nuclei. Fractions of images are enlarged for better visualization. **B.** Fluorescent immunostaining of ANXA2 in Panc10.05 cells either uninfected or infected with lentivirus expressing wild-type ANXA2, lentivirus expressing Y23A mutated ANXA2 or lentivirus expressing Y23E mutated ANXA2. FITC images or overlapped images of FITC and DAPI staining are shown as indicated. Note that immunostaining of ANXA2 detected both exogenous and endogenous ANXA2. In cells infected with the tyrosine site loss variant LV-ANXA2^Y23A^, even endogenous ANXA2 no longer localized to the cell surface, suggesting that ANXA2^Y23A^ has a dominant negative effect (**Figure S5)**. **C.** FLAG-tagged ANXA2 expression in Panc10.05 cells transfected with the pcDNA-based plasmid carrying ANXA2^WT^-FLAG, the plasmid carrying ANXA2^Y23A^-FLAG, or the plasmid carrying ANXA2^Y23E^-FLAG. Membrane fractions (M) and cytoplasmic fractions (C) were isolated by biochemical fractionation and blotted with mouse anti-GST antibodies as a quality control. The result shows that the membrane fractions are not contaminated by cytoplasmic protein. **D.** *In vitro* invasion of Panc10.05 cells transfected with the pcDNA-based plasmid carrying ANXA2^WT^-FLAG (lane 1) or the plasmid carrying ANXA2^Y23A^-FLAG (lane 2). Results of duplicate experiments are shown.
(TIF)

Figure S5 **The ANXA2-Y23A mutant demonstrates a dominant negative effect.** **A.** Panc10.05 cells infected with the lentivirus expressing wild-type ANXA2 (lanes 1,2) or the lentivirus expressing Y23A mutated ANXA2 (lanes 3–5) were fractionated into membrane (M, lanes 1,2,4,5) and cytoplasmic fractions (C, lane 3). The fractions were immunoprecipated by either rabbit anti-ANXA2 antibodies or mouse anti-p11 antibodies as indicated and immunoblotted with anti-ANXA2 antibodies and anti-p11 antibodies, respectively. Note that ANXA2 and p11 can be co-immunoprecipated from the membrane fraction of the cells exogenously expressing ANXA2-WT and from the cytoplasmic fraction of the cells exogenously expressing ANXA2-Y23A, suggesting that ANXA2-Y23A does not affect the complex of ANXA2 and p11 in the cytosol. However, neither ANXA2 nor p11 can be detected in the membrane fraction of cells exogenously expressing ANXA2-Y23A although these cells should still have endogenous expression of the wild-type ANXA2. Therefore, this result suggests that exogenous ANXA2-Y23A may have sequestered p11 in the cytosol. **B.** Whole cell extracts from Panc10.05 cells either uninfected (lane 1), infected with lentivirus expressing wild-type ANXA2 (lane 2), lentivirus expressing Y23A mutated ANXA2 (lane 3), or lentivirus expressing Y23E mutated ANXA2 (lane 4), were analyzed by western blot with rabbit anti-ANXA2 antibodies and mouse anti-p11 antibodies. This result shows that the total ANXA2 expression is increased in the cells exogenously expressing ANXA2 as compared with that in the parental cells, whereas the expression level of p11 remains to be the same. This result further suggests that cytoplasmic-localized ANXA2-Y23A has the potential to sequester all the p11 proteins in the cytosol because it is overexpressed and more abundant than the endogenous wild-type ANXA2. Therefore, the reason for the observed dominant negative effect of overexpressed ANXA2-Y23A is likely due to little p11 being available to bind endogenous wild-type ANXA2 which depends on p11 for its translocation [16]. The same experiments were done with mouse Panc02 cells and similar results were obtained.
(TIF)

Figure S6 **Liver metastases model of mouse PDAs.** Schema showing the hemisplenectomy model for establishing PDA liver metastases. Mouse PDA cells injected into the hemi-spleen of syngeneic mice form tumors at the splenic bed and metastases in the liver.
(TIF)

Figure S7 **Histological analysis of tumors formed in the liver metastases model.** Shown are representative results of IHC analysis evaluating ANXA2 expression using polyclonal anti-ANXA2 antibodies to stain tumors formed at the splenic bed (left panels) and in the liver (right panels). Upper panels received control shRNA and lower panels received *ANXA2* shRNA. All panels, 10x amplification. Mice were examined macroscopically; and multiple sections of livers and splenic bed injection sites were examined microscopically with H&E staining. All mice in both groups had tumors at the splenic bed injection site. These tumors were likely formed by PDA cells left behind during the splenic injection. Sizes of these tumors were difficult to measure as they adhered and/or infiltrated the remaining spleen. However, tumors that formed at the splenic bed in mice of the *ANXA2* shRNA group appeared to be larger and more prominent than those of the control group. It is possible that the prolonged survival in these mice allowed continued locoregional growth when compared with the tumors in the control mice. IHC analyses were performed on the liver metastases and the locoreginal tumors that formed at the splenic bed to evaluate ANXA2 expression and localization. As shown in this figure, PDA cells in tumors excised from the control mice that formed at the splenic bed (panel **a**) and that metastasized to the liver (panel **b**) stained positive for cell surface ANXA2. In contrast, the majority of PDA cells expressing *ANXA2* shRNA in tumors that formed at the splenic bed failed to stain for ANXA2 (panel **c**), consistent with the effect of shRNA. It is not surprising to see a few ANXA2 positive PDA cells (representatives indicated by arrows) because RNA interference is not able to knock down gene expression completely. Panel **d**, arrow indicates a micro-metastasis that expresses ANXA2. Of note, 5 of the 17 mice in the *ANXA2* shRNA group were found to have microscopic metastases in their liver on H&E staining. These micro-metastatic tumors stained positively for cell surface ANXA2, supporting ANXA2 mediated metastasis formation likely due to the incomplete knockdown of ANXA2 with shRNA.
(TIF)

Figure S8 **Histological analysis of metastases formed in the orthotopic PDA model.** The orthotopic PDA model was perfomed as previously described [25]. Briefly, 1×10^6^ mouse PDA cells were injected s.c. into syngeneic female C57Bl/6 mice. After 2 to 4 weeks, the s.c. tumors were harvested and cut into cubes of ∼1 mm^3^. New syngeneic female C57Bl/6mice, ages 8 to 10 weeks, were anesthetized. The abdomen was opened via a subcostal left incision of 1 cm. A small pocket was prepared inside the pancreas using microscissors, into which one piece of the s.c. tumor was implanted. The incision in the pancreas was closed with a suture. The abdominal wall was sutured, and the skin adapted using wound clips. For the first experiment, 5 mice were implanted with Panc02 tumors infected with control lentivirus and 8 mice with *ANXA2* shRNA. On day 25 following implantation, some mice in the control group were found dead. Therefore, remaining mice in both groups were euthanized for necropsy. For the second experiment, an additional 6 mice were implanted with tumors infected with control lentivirus and 7 mice with *ANXA2* shRNA. On day 21 following implantation, all the mice in both groups were euthanized while they were all still alive. 11/11 mice in the control group developed severe peritoneal dissemination of large implants. Although 10/15 mice in the *ANXA2* shRNA group developed peritoneal metastases, they were less extensive, smaller peritoneal metastases. **A.** Shown are representative H&E staining sections of peritoneal metastases, indicated by arrows. Left panels: control shRNA group; right panels: *ANXA2* shRNA group. Left and right lower panels: enlarged areas of peritoneal metastases. **B.** At the time of necropsy, PDAs at the primary implantation site were measured and did not differ significantly between the two groups (p = 0.248). **C.** The comparison of the sizes of peritoneal metastases between the two groups in the second experiment is shown. The sizes of peritoneal implants differs significantly between the two groups (p = 0.0012). The largest metastasis from each mouse was chosen for paraffin-embedding and for measurement. Because mice in the ANXA2 group developed very small peritoneal metastases, all measurements were done on the microscope-scanned, H&E stained, tissue slides. **D.** Representative results of immunohistochemistry analysis with polyclonal anti-ANXA2 antibodies of tumors formed in the pancreas and peritoneal metastases are shown. Subpanels (a),(c),(d), 10x amplification; subpanel (b), 4x amplification. In subpanel (d), arrow indicates a small peritoneal metastasis. Note that the small peritoneal tumors from the *ANXA2* shRNA group stained positive for cell surface ANXA2, suggesting that they were formed by cells that still expressed ANXA2 due to incomplete knockdown by *ANXA2* shRNA.
(TIF)
