# Supplementary material for: Tyrosine 23 Phosphorylation-Dependent Cell-Surface Localization of Annexin A2 Is Required for Invasion and Metastases of Pancreatic Cancer
Source: PLoS One. 2011 Apr 29;6(4):e19390. doi: 10.1371/journal.pone.0019390 (PMC3084841; doi:10.1371/journal.pone.0019390)
Supplement: Table S1 — Cell Surface Localization of Annexin A2 in Cells with Different Invasive Capacities. (PDF) [file pone.0019390.s001.pdf]

Table S1. Cell Surface Localization of Annexin A2 in Cells with Different Invasive Capacities

	Cell lines	Cell Surface Localization of Annexin A2
High invasive capacity	Panc10.05	+
	Panc01.28	+
	Panc2.8	+
	Panc2.03	+
	Panc4.03	+
	TS0129	+
	Panc2.13	+
	Panc2.43	+
Low invasive capacity	Panc3.11	-
	Panc9.3.96	-
	Panc6.03	+
	Fibroblast Cells	-
