# Supplementary figures and images for: Tyrosine 23 Phosphorylation-Dependent Cell-Surface Localization of Annexin A2 Is Required for Invasion and Metastases of Pancreatic Cancer
Source: PLoS One. 2011 Apr 29;6(4):e19390. doi: 10.1371/journal.pone.0019390 (PMC3084841; doi:10.1371/journal.pone.0019390)

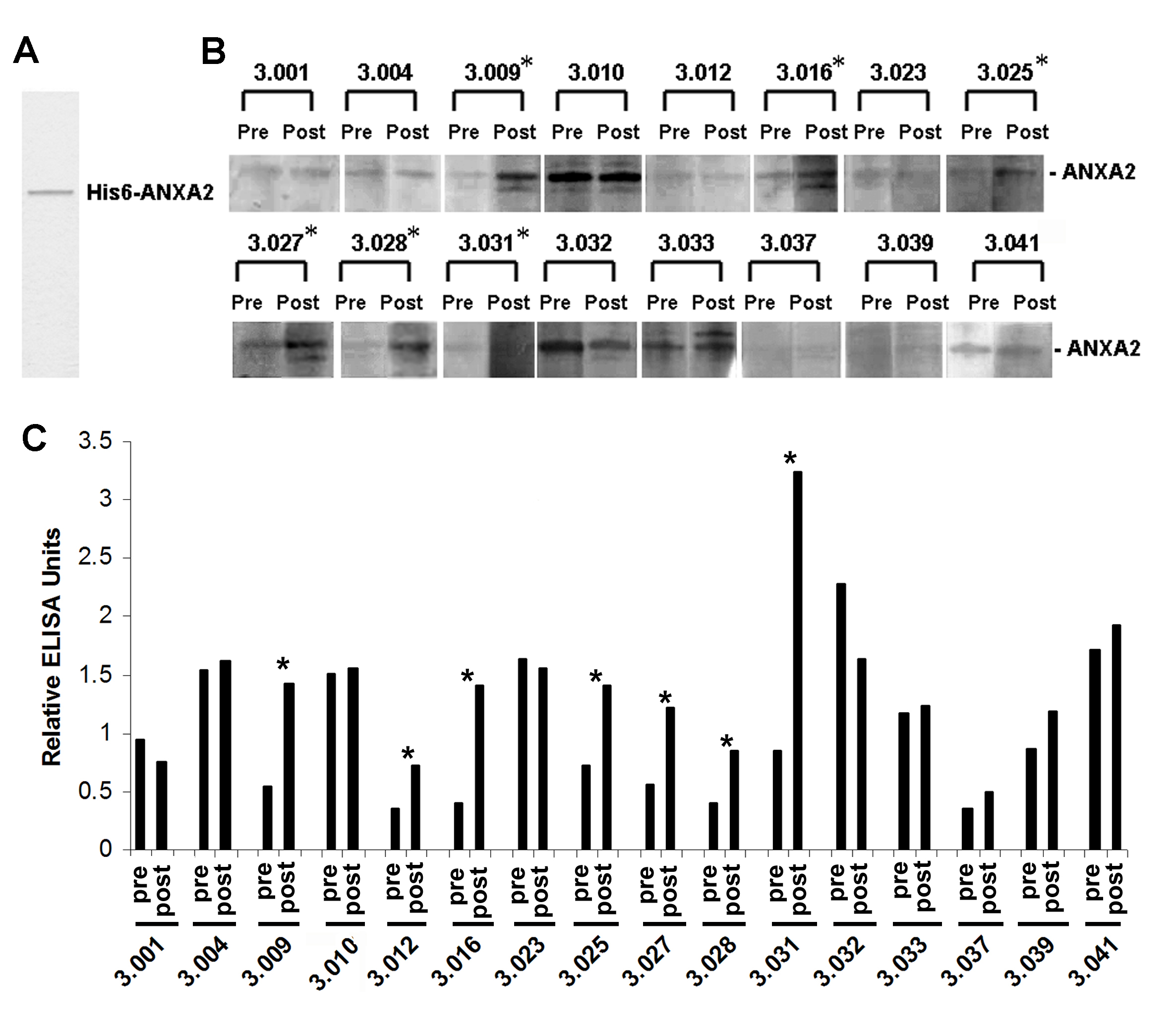

Supplement: Figure S1 — PDA patients demonstrate vaccine induced ANXA2-specific serologic responses. A. Purified recombinant His6-tagged ANXA2 (His6-ANXA2) on a SDS-PAGE gel stained with coommassie blue. B. Purified His6 tagged ANXA2 on a SDS-PAGE gel was western-blotted by pre- and post-vaccination sera. Patients marked by * had antibody induction, which was manifested by stronger signals of ANXA2 on western blot with post-vaccination versus pre-vaccination sera. C. Pre- and post-vaccination sera from patients were tested for the presence of anti-ANXA2 antibodies by ELISA using purified recombinant ANXA2-coated plates. Positive antibody induction, which was manifested by a more than 2-fold increase in antibody reaction, was marked by *. (TIF) [file pone.0019390.s002.tif]

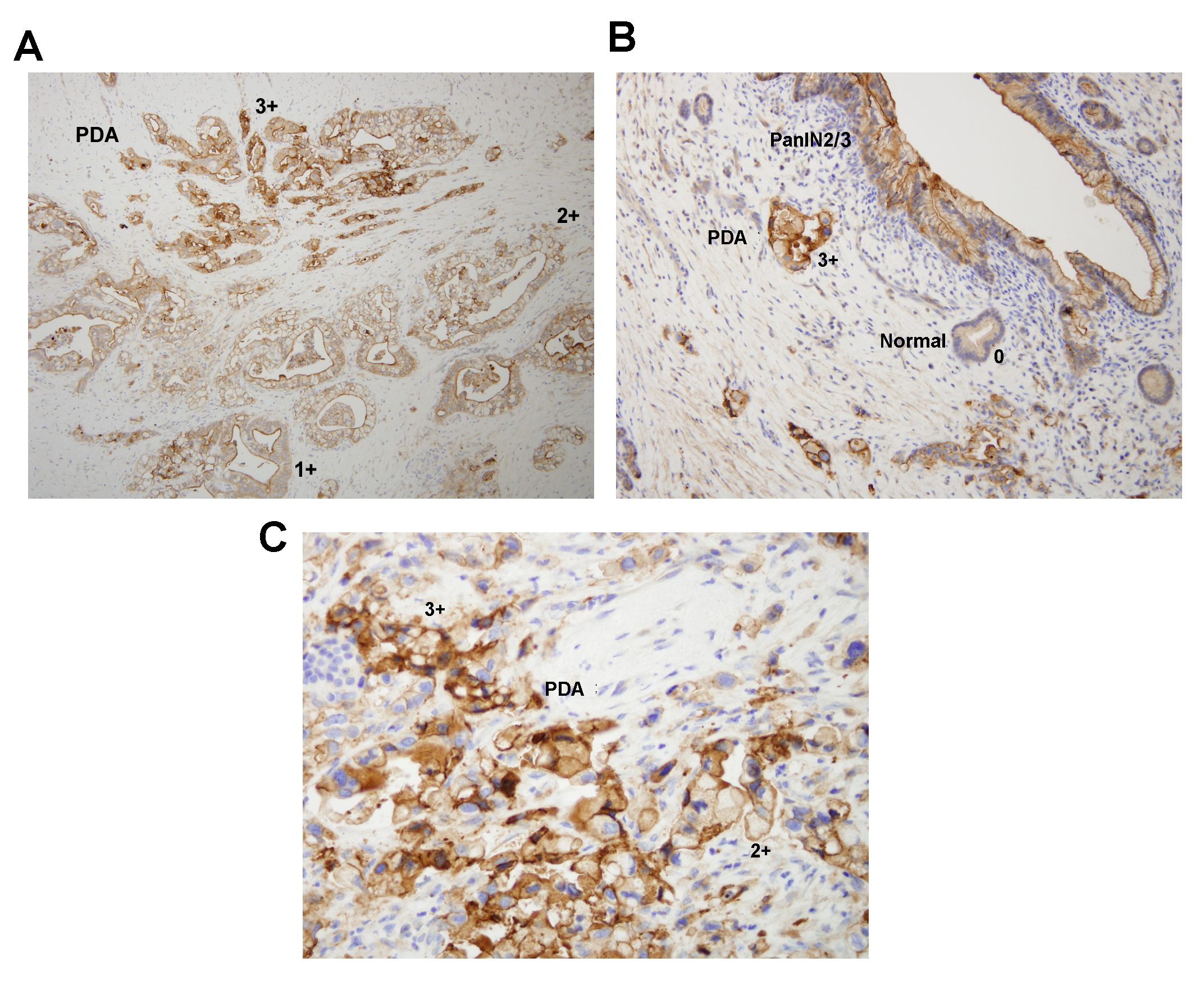

Supplement: Figure S2 — Cell surface expression of ANXA2 is increased in the majority of human PDAs. The pattern of ANXA2 expression was analyzed by IHC in 52 of 60 resected tumors from patients treated in a Phase II study for whom specimens were available for staining. Representative IHC staining of ANXA2 in these human PDAs is shown (panels A, B, C). Cell-surface expression of ANXA2 was semiquantitated using a score of 0 to 3, with a score of 0 representing no staining and a score of 3 representing the strongest staining. Normal pancreatic duct, PanINs and PDA are indicated. Cytoplasmic and luminal staining was excluded from scoring. Shown is the intensity of ANXA2 expression on the cell surface. PDA cells vary in their ANXA2 expression level within the same tumor tissue (panel C). To account for expression variability within each specimen, the percentage of PDA cells at each score level was estimated and the average score of each PDA tissue was calculated by multiplying each score by their percentages (see Supporting Information Materials and Methods). As expected, none of the normal appearing ductal epithelial cells within the resected tumor masses express 3+ ANXA2 and few express 2+ ANXA2 (panel B). An average score of 1.5 or above was considered representative of increased cell surface expression of ANXA2 in the tumor tissue. (TIF) [file pone.0019390.s003.tif]

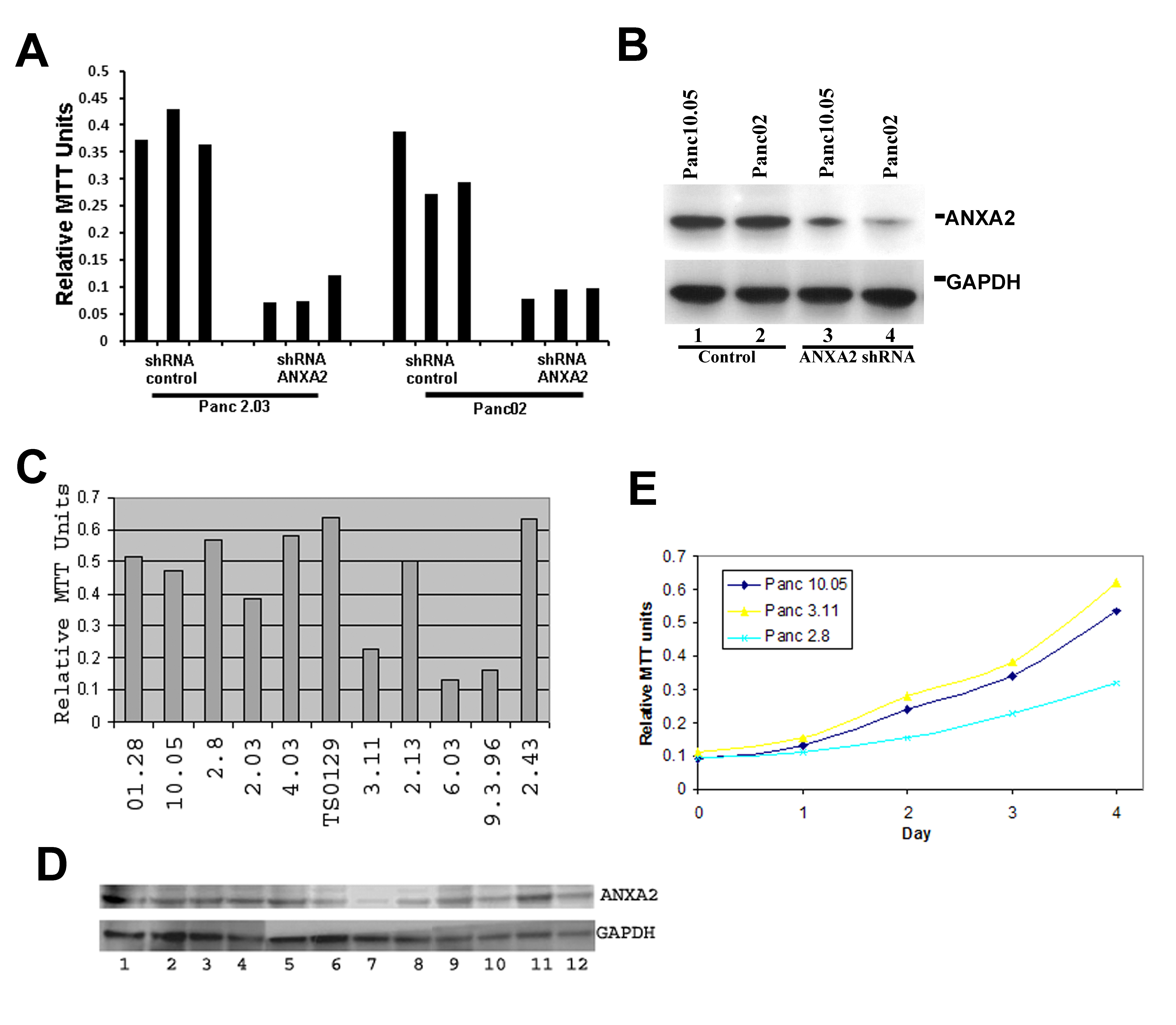

Supplement: Figure S3 — ANXA2 shRNA inhibits the invasion capacity of multiple human and mouse PDA cell lines and PDA cell invasion potentials vary among different PDA cell lines and do not correlate with their proliferative rates. A. Human Panc2.03 PDA cells or mouse Panc02 PDA cells were infected with lentivirus carrying the shRNA specific for human or mouse ANXA2 or lentivirus carrying the control shRNA. Invaded cells were measured by MTT assays and normalized by total cell numbers. Triplicate experiments were done for control shRNA and ANXA2 shRNA, respectively. B. Panc10.05 (lanes 1,3) or Panc02 cells (lanes 2,4) infected with lentivirus either carrying shRNA specific for human or mouse ANXA2 knockdown (lanes 3 and 4, respectively) or carrying control shRNA (lanes 1,2) were sorted for GFP-positive cells by FACS with one aliquot of cells saved for analysis of ANXA2 expression prior to each experiment. A representative western blot using the rabbit anti-ANXA2 antibody and the rabbit anti-GAPDH antibody (control) is shown. C. A panel of PDA cell lines derived from primary resected tumors were evaluated in an in vitro invasion assay. Of 11 PDA cell lines tested, 8 have higher and 3 have lower invasion capacity (Table S1). Shown are average MTT units on three parallel experiments normalized to total cell numbers. D. Expression of ANXA2 in each PDA cell line demonstrated by immunoblot analyses with anti-ANXA2 polyclonal antibody. Lanes 1–11 correspond to human PDA cell lines: Panc01.28, Panc10.05, Panc2.8, Panc2.03, Panc4.03, PancTS0129, Panc3.11, Panc2.13, Panc6.03, Panc9.3.96, and Panc2.43, respectively; lane 12, human pancreatic para-cancerous fibroblast cells. Expression of ANXA2 is slightly lower in cells with lower invasion capacity and slightly higher in those with higher invasion capacity, suggesting that over expression of ANXA2 in PDAs may contribute to, but does not entirely explain the range of invasion potential of these PDA cell lines. E. Growth curves of selected PDA cell lin [file pone.0019390.s004.tif]

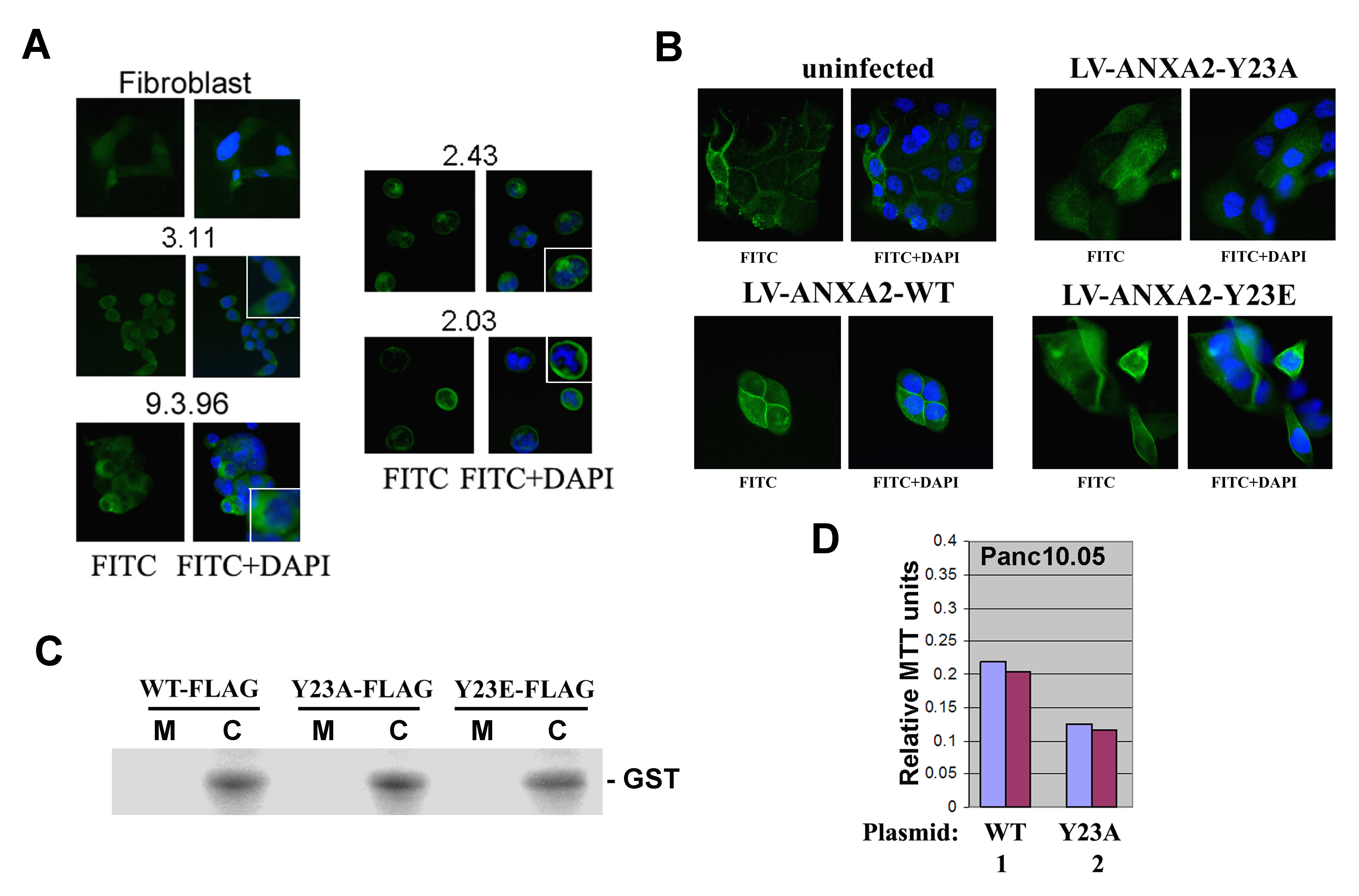

Supplement: Figure S4 — PDA cell invasion potential correlates with ANXA2 surface localization on PDA cells and PDA cells expressing the exogenous ANXA2-Y23A mutant have reduced surface localization and invasion potential in vitro . A. Fluorescent immunostaining shows predominant cell surface localization of ANXA2 in representative cells with higher invasion capacity (Panc2.43, Panc2.03), but not in cells with lower invasion capacity (human fibroblast, Panc3.11, Panc9.3.96). See supplemental Figure 6 for Panc10.05 cells. FITC indicates the images of immunostaining with rabbit anti-ANXA2 polyclonal antibody and FITC-conjugated secondary antibody. FITC+DAPI indicates the overlapped images of FITC staining of ANXA2 and DAPI staining of nuclei. Fractions of images are enlarged for better visualization. B. Fluorescent immunostaining of ANXA2 in Panc10.05 cells either uninfected or infected with lentivirus expressing wild-type ANXA2, lentivirus expressing Y23A mutated ANXA2 or lentivirus expressing Y23E mutated ANXA2. FITC images or overlapped images of FITC and DAPI staining are shown as indicated. Note that immunostaining of ANXA2 detected both exogenous and endogenous ANXA2. In cells infected with the tyrosine site loss variant LV-ANXA2Y23A, even endogenous ANXA2 no longer localized to the cell surface, suggesting that ANXA2Y23A has a dominant negative effect (Figure S5). C. FLAG-tagged ANXA2 expression in Panc10.05 cells transfected with the pcDNA-based plasmid carrying ANXA2WT-FLAG, the plasmid carrying ANXA2Y23A-FLAG, or the plasmid carrying ANXA2Y23E-FLAG. Membrane fractions (M) and cytoplasmic fractions (C) were isolated by biochemical fractionation and blotted with mouse anti-GST antibodies as a quality control. The result shows that the membrane fractions are not contaminated by cytoplasmic protein. D. In vitro invasion of Panc10.05 cells transfected with the pcDNA-based plasmid carrying ANXA2WT-FLAG (lane 1) or the plasmid carrying ANXA2Y23A-FLAG (lane 2). Results of duplicate experim [file pone.0019390.s005.tif]

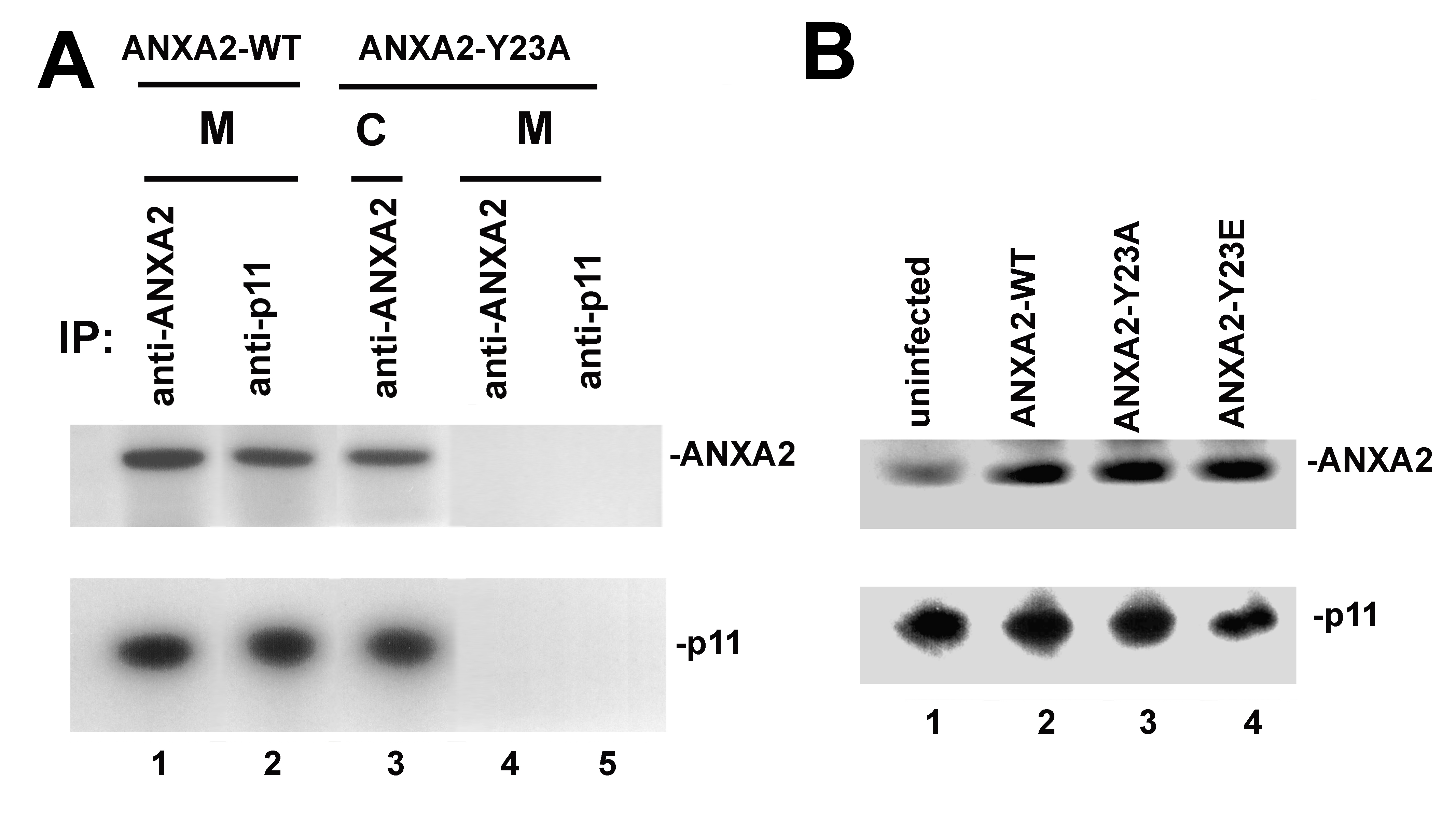

Supplement: Figure S5 — The ANXA2-Y23A mutant demonstrates a dominant negative effect. A. Panc10.05 cells infected with the lentivirus expressing wild-type ANXA2 (lanes 1,2) or the lentivirus expressing Y23A mutated ANXA2 (lanes 3–5) were fractionated into membrane (M, lanes 1,2,4,5) and cytoplasmic fractions (C, lane 3). The fractions were immunoprecipated by either rabbit anti-ANXA2 antibodies or mouse anti-p11 antibodies as indicated and immunoblotted with anti-ANXA2 antibodies and anti-p11 antibodies, respectively. Note that ANXA2 and p11 can be co-immunoprecipated from the membrane fraction of the cells exogenously expressing ANXA2-WT and from the cytoplasmic fraction of the cells exogenously expressing ANXA2-Y23A, suggesting that ANXA2-Y23A does not affect the complex of ANXA2 and p11 in the cytosol. However, neither ANXA2 nor p11 can be detected in the membrane fraction of cells exogenously expressing ANXA2-Y23A although these cells should still have endogenous expression of the wild-type ANXA2. Therefore, this result suggests that exogenous ANXA2-Y23A may have sequestered p11 in the cytosol. B. Whole cell extracts from Panc10.05 cells either uninfected (lane 1), infected with lentivirus expressing wild-type ANXA2 (lane 2), lentivirus expressing Y23A mutated ANXA2 (lane 3), or lentivirus expressing Y23E mutated ANXA2 (lane 4), were analyzed by western blot with rabbit anti-ANXA2 antibodies and mouse anti-p11 antibodies. This result shows that the total ANXA2 expression is increased in the cells exogenously expressing ANXA2 as compared with that in the parental cells, whereas the expression level of p11 remains to be the same. This result further suggests that cytoplasmic-localized ANXA2-Y23A has the potential to sequester all the p11 proteins in the cytosol because it is overexpressed and more abundant than the endogenous wild-type ANXA2. Therefore, the reason for the observed dominant negative effect of overexpressed ANXA2-Y23A is likely due to little p11 being available to bind en [file pone.0019390.s006.tif]

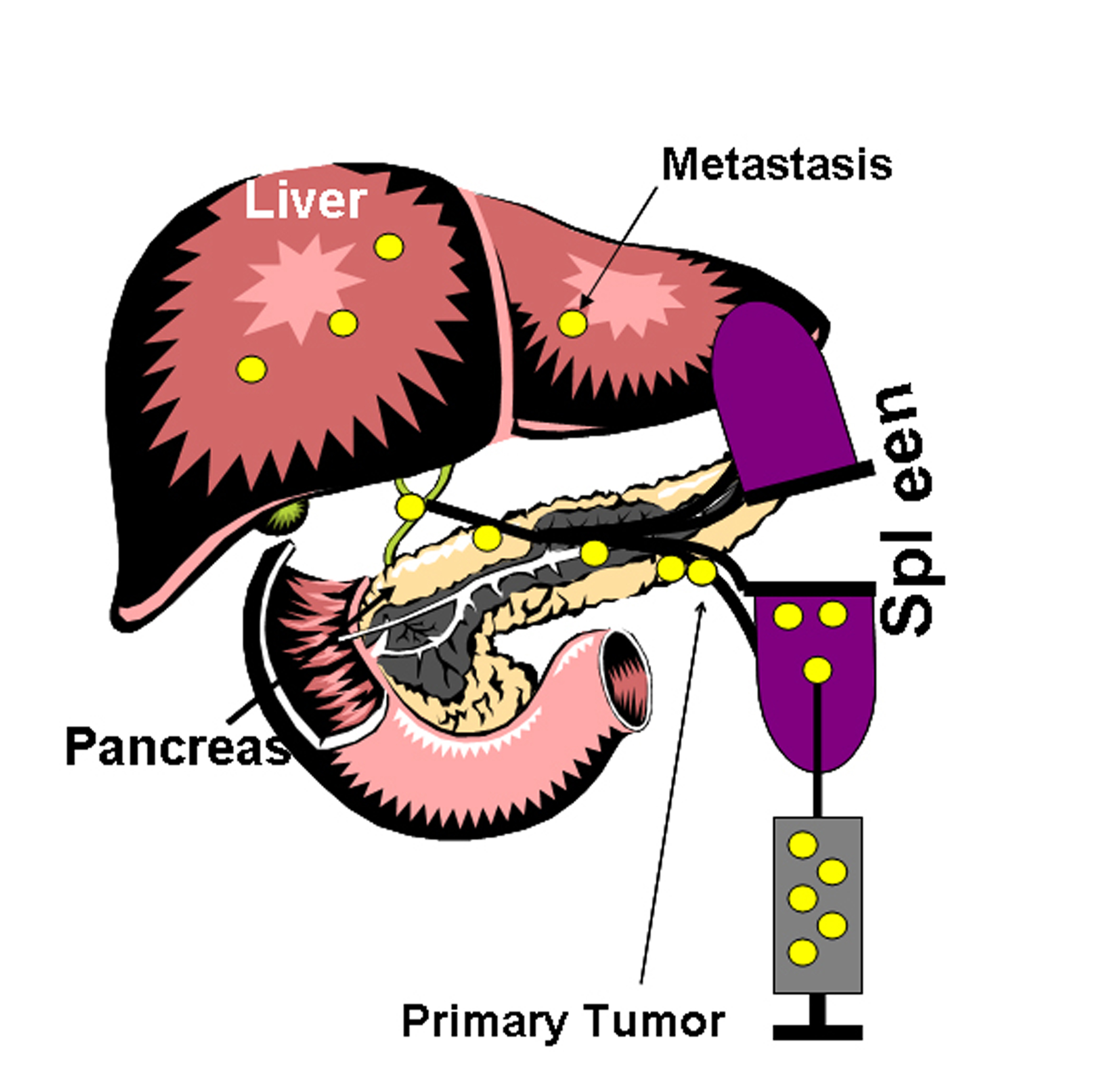

Supplement: Figure S6 — Liver metastases model of mouse PDAs. Schema showing the hemisplenectomy model for establishing PDA liver metastases. Mouse PDA cells injected into the hemi-spleen of syngeneic mice form tumors at the splenic bed and metastases in the liver. (TIF) [file pone.0019390.s007.tif]

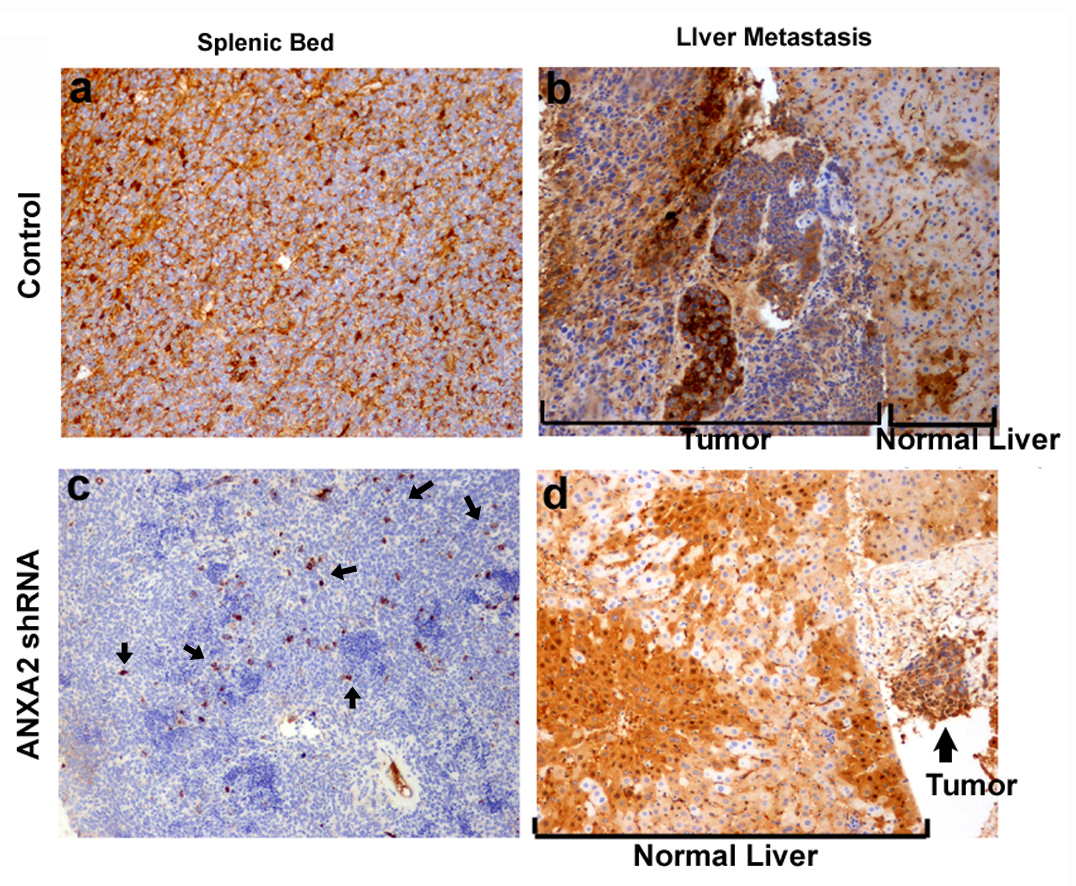

Supplement: Figure S7 — Histological analysis of tumors formed in the liver metastases model. Shown are representative results of IHC analysis evaluating ANXA2 expression using polyclonal anti-ANXA2 antibodies to stain tumors formed at the splenic bed (left panels) and in the liver (right panels). Upper panels received control shRNA and lower panels received ANXA2 shRNA. All panels, 10x amplification. Mice were examined macroscopically; and multiple sections of livers and splenic bed injection sites were examined microscopically with H&E staining. All mice in both groups had tumors at the splenic bed injection site. These tumors were likely formed by PDA cells left behind during the splenic injection. Sizes of these tumors were difficult to measure as they adhered and/or infiltrated the remaining spleen. However, tumors that formed at the splenic bed in mice of the ANXA2 shRNA group appeared to be larger and more prominent than those of the control group. It is possible that the prolonged survival in these mice allowed continued locoregional growth when compared with the tumors in the control mice. IHC analyses were performed on the liver metastases and the locoreginal tumors that formed at the splenic bed to evaluate ANXA2 expression and localization. As shown in this figure, PDA cells in tumors excised from the control mice that formed at the splenic bed (panel a) and that metastasized to the liver (panel b) stained positive for cell surface ANXA2. In contrast, the majority of PDA cells expressing ANXA2 shRNA in tumors that formed at the splenic bed failed to stain for ANXA2 (panel c), consistent with the effect of shRNA. It is not surprising to see a few ANXA2 positive PDA cells (representatives indicated by arrows) because RNA interference is not able to knock down gene expression completely. Panel d, arrow indicates a micro-metastasis that expresses ANXA2. Of note, 5 of the 17 mice in the ANXA2 shRNA group were found to have microscopic metastases in their liver on H&E staining. These [file pone.0019390.s008.tif]

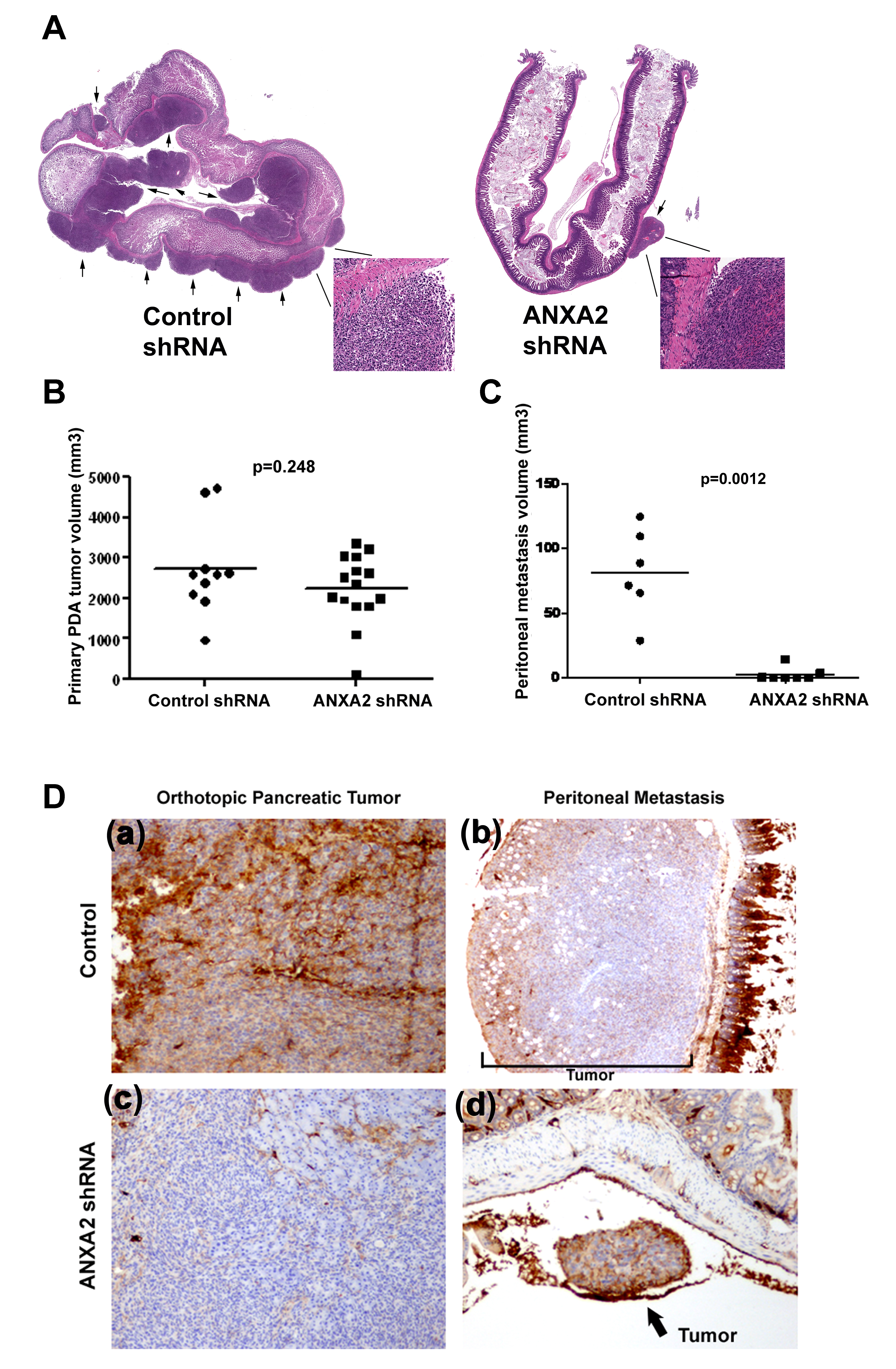

Supplement: Figure S8 — Histological analysis of metastases formed in the orthotopic PDA model. The orthotopic PDA model was perfomed as previously described [25]. Briefly, 1×10^6^ mouse PDA cells were injected s.c. into syngeneic female C57Bl/6 mice. After 2 to 4 weeks, the s.c. tumors were harvested and cut into cubes of ∼1 mm3. New syngeneic female C57Bl/6mice, ages 8 to 10 weeks, were anesthetized. The abdomen was opened via a subcostal left incision of 1 cm. A small pocket was prepared inside the pancreas using microscissors, into which one piece of the s.c. tumor was implanted. The incision in the pancreas was closed with a suture. The abdominal wall was sutured, and the skin adapted using wound clips. For the first experiment, 5 mice were implanted with Panc02 tumors infected with control lentivirus and 8 mice with ANXA2 shRNA. On day 25 following implantation, some mice in the control group were found dead. Therefore, remaining mice in both groups were euthanized for necropsy. For the second experiment, an additional 6 mice were implanted with tumors infected with control lentivirus and 7 mice with ANXA2 shRNA. On day 21 following implantation, all the mice in both groups were euthanized while they were all still alive. 11/11 mice in the control group developed severe peritoneal dissemination of large implants. Although 10/15 mice in the ANXA2 shRNA group developed peritoneal metastases, they were less extensive, smaller peritoneal metastases. A. Shown are representative H&E staining sections of peritoneal metastases, indicated by arrows. Left panels: control shRNA group; right panels: ANXA2 shRNA group. Left and right lower panels: enlarged areas of peritoneal metastases. B. At the time of necropsy, PDAs at the primary implantation site were measured and did not differ significantly between the two groups (p = 0.248). C. The comparison of the sizes of peritoneal metastases between the two groups in the second experiment is shown. The sizes of peritoneal implants differs significantl [file pone.0019390.s009.tif]
